# The Impact of mHealth Interventions: Systematic Review of Systematic Reviews

**DOI:** 10.2196/mhealth.8873

**Published:** 2018-01-17

**Authors:** Milena Soriano Marcolino, João Antonio Queiroz Oliveira, Marcelo D'Agostino, Antonio Luiz Ribeiro, Maria Beatriz Moreira Alkmim, David Novillo-Ortiz

**Affiliations:** ^1^ Medical School and Telehealth Center, University Hospital Universidade Federal de Minas Gerais Belo Horizonte Brazil; ^2^ Pan American Health Organization Washington, DC United States

**Keywords:** telemedicine, medical informatics, mobile phones

## Abstract

**Background:**

Mobile phone usage has been rapidly increasing worldwide. mHealth could efficiently deliver high-quality health care, but the evidence supporting its current effectiveness is still mixed.

**Objective:**

We performed a systematic review of systematic reviews to assess the impact or effectiveness of mobile health (mHealth) interventions in different health conditions and in the processes of health care service delivery.

**Methods:**

We used a common search strategy of five major scientific databases, restricting the search by publication date, language, and parameters in methodology and content. Methodological quality was evaluated using the Measurement Tool to Assess Systematic Reviews (AMSTAR) checklist.

**Results:**

The searches resulted in a total of 10,689 articles. Of these, 23 systematic reviews (371 studies; more than 79,665 patients) were included. Seventeen reviews included studies performed in low- and middle-income countries. The studies used diverse mHealth interventions, most frequently text messaging (short message service, SMS) applied to different purposes (reminder, alert, education, motivation, prevention). Ten reviews were rated as low quality (AMSTAR score 0-4), seven were rated as moderate quality (AMSTAR score 5-8), and six were categorized as high quality (AMSTAR score 9-11). A beneficial impact of mHealth was observed in chronic disease management, showing improvement in symptoms and peak flow variability in asthma patients, reducing hospitalizations and improving forced expiratory volume in 1 second; improving chronic pulmonary diseases symptoms; improving heart failure symptoms, reducing deaths and hospitalization; improving glycemic control in diabetes patients; improving blood pressure in hypertensive patients; and reducing weight in overweight and obese patients. Studies also showed a positive impact of SMS reminders in improving attendance rates, with a similar impact to phone call reminders at reduced cost, and improved adherence to tuberculosis and human immunodeficiency virus therapy in some scenarios, with evidence of decrease of viral load.

**Conclusions:**

Although mHealth is growing in popularity, the evidence for efficacy is still limited. In general, the methodological quality of the studies included in the systematic reviews is low. For some fields, its impact is not evident, the results are mixed, or no long-term studies exist. Exceptions include the moderate quality evidence of improvement in asthma patients, attendance rates, and increased smoking abstinence rates. Most studies were performed in high-income countries, implying that mHealth is still at an early stage of development in low-income countries.

## Introduction

Mobile phone usage has been rapidly increasing worldwide [1,2]. In many high-income countries, mobile phone subscriptions exceed the population, and in many low- and middle-income countries, this number is expanding faster than other infrastructures [2]. Mobile technology’s mobility, instantaneous access, and direct communication allow for faster transfer of health information, which in turn supports medical and public health practices. These characteristics define mobile health (mHealth). mHealth could transform the worldwide delivery of health services, especially in low- and middle-income countries. This includes simple apps and complex technologies including voice, text messaging (short message service, SMS), multimedia message service, Bluetooth technology, and others [3].

mHealth is increasingly being used (1) for patient communication, monitoring, and education, (2) to reduce the burden of diseases linked with poverty, (3) to improve access to health services, clinical diagnosis, and treatment adherence, and (4) for chronic disease management [4-6].

It is commonly stated that mHealth effectively improves the quality of care and that it can quickly be adapted on a large scale and at low cost, but evidence regarding its effectiveness and cost-effectiveness is still lacking in different areas. As the evidence in this field is consistently growing, many systematic reviews have already been performed. A thorough review of available evidence is essential to guide clinical and health policy decisions. Consequently, complex reviews, which may assess multiple interventions, different or distinct populations, and different outcomes may adequately support health policy decision making in this context [7]. Therefore, the objective of this study was to perform a systematic review of systematic reviews that assessed the effectiveness of mHealth interventions in different health conditions and in the processes of health care service delivery, in order to investigate for which areas there is evidence and which areas still require further studies.

## Methods

This study is a systematic review of systematic reviews and is part of a series of four reviews that assessed the impact to telehealth strategies in different health conditions and in health care service delivery. The study was conducted in accordance with the Preferred Reporting Items for Systematic Reviews and Meta-Analyses (PRISMA) statement and the methodological considerations when using existing systematic reviews [7].

### Search Strategy and Inclusion Criteria

A literature search was performed using MEDLINE (accessed by PubMed), IEEE Xplore Digital Library, Cochrane (Cochrane Database of Systematic Reviews, Cochrane Central Register of Controlled Trials, Cochrane Methodology Register, Database of Abstracts of Reviews of Effects, National Health Service Economic Evaluation Database), *Literatura Latino-Americana e do Caribe em Ciências da Saúde* (LILACS), and *Indice Bibliográfico Español de Ciencias de la Salud* (IBECS) in November 2015. Cochrane, LILACS, and IBECS were assessed by Virtual Health Library (*Biblioteca Virtual em Saúde*). The search was restricted to studies in humans, publication date (from January 1, 2000, up to the search data), and publication language (English, French, Spanish, Italian, and Portuguese).

We used a common search strategy and allocated relevant studies to their respective reviews before assessing their risk of bias and extracting data. The search strategies for each database are given in Multimedia Appendix 1. All studies were included in the software StArt (State of the Art through Systematic Review) [8]. In this software, different combinations of the terms “systematic” and “review” identified systematic reviews by title or abstract.

An additional search using the same terms and parameters was performed in February 2016. The new search was more specific to make assessment manageable and was supplemented by a manual search of reference lists [9].

### Study Selection

Systematic reviews covering the effectiveness or cost-benefit analysis of eHealth interventions were included. Exclusion criteria were (1) studies about feasibility, user acceptance, or usability, (2) studies that assessed “perceived benefits,” and (3) nonsystematic reviews.

Initial screening was based on titles and abstracts, and articles were independently evaluated. Abstracts lacking information were retrieved for full-text evaluation. Subsequently, 2 investigators independently evaluated full-text articles and determined eligibility. Authorship, journal, or years were not blinded.

### Data Extraction and Quality Assessment

Five investigators conducted data extraction following standardized criteria, and results were reviewed by 2 senior researchers. The following data were extracted: journal, publication year, databases searched, time period, setting/scenario, theme/specialty, objective, intervention type, number of studies, total number and countries of patients, study design, whether a review of systematic reviews or meta-analysis was performed, outcomes, main results, lessons and barriers for implementation, and main limitations. For cost analysis, the type and the perspective (ie, patient, health care provider, and/or society) were also extracted. Studies were evaluated using the Measurement Tool to Assess Systematic Reviews (AMSTAR) checklist for assessing methodological quality [10].

## Results

**Figure 1:**
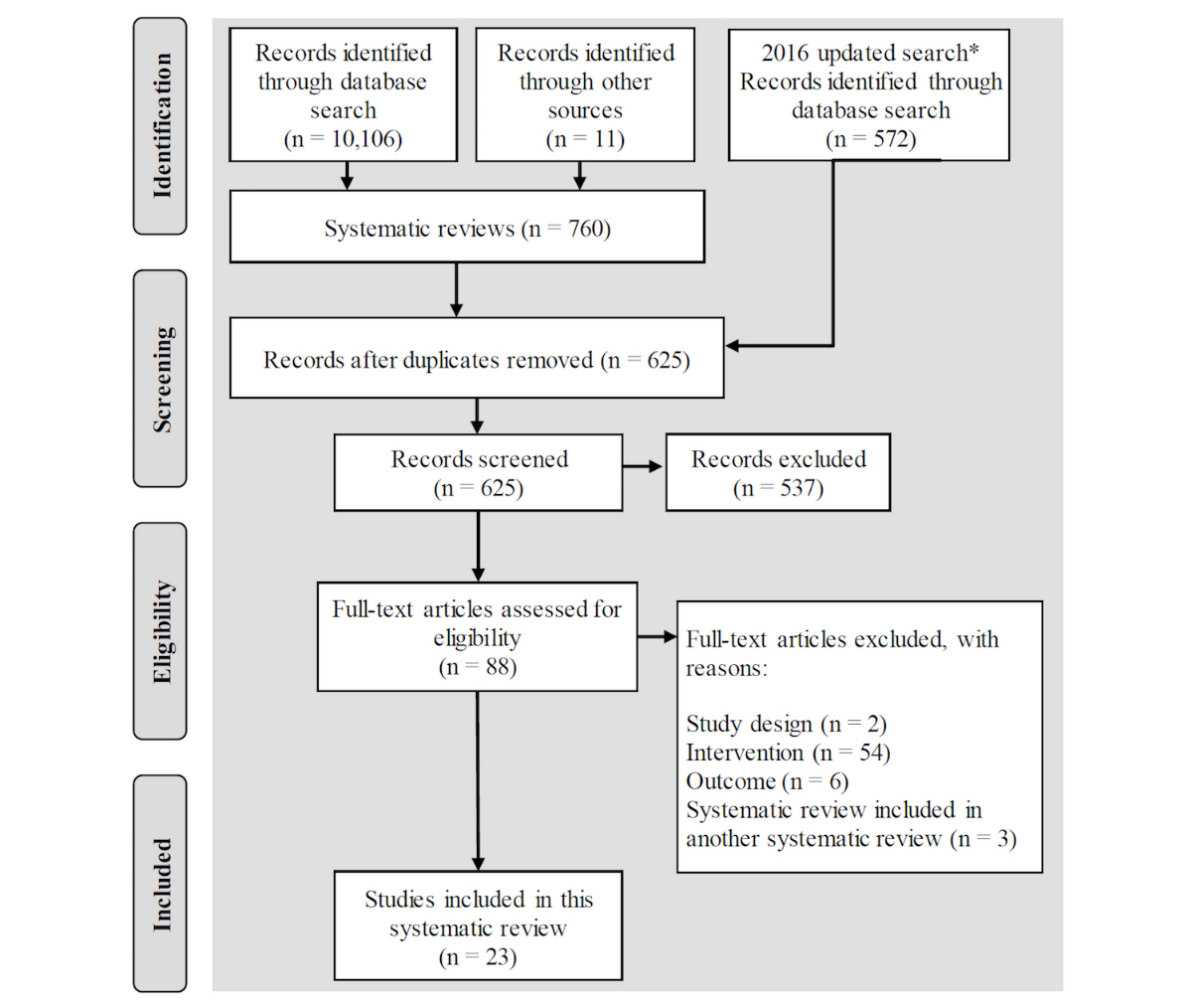
Flow of information through the different phases of the systematic review. Asterisk indicates that this search was limited to systematic reviews.

A flow diagram of literature search and study selection results is shown in Figure 1. The database first search resulted in 10,106 articles, the updated search resulted in 572 articles, and 11 studies were found from additional sources. After exclusion of duplicates, 625 articles were screened and 537 were excluded. Full text of 88 eligible articles was reviewed. Out of these, 62 were excluded for not meeting the criteria relating to study type, intervention, or outcome. Three studies [5,11,12] were excluded for being included in a systematic review of systematic reviews that was included in this manuscript, to avoid duplication. The 23 studies remaining were included in this systematic review.

### Descriptive Analysis of the Systematic Reviews

#### General Characteristics of Reviewed Papers

The 23 systematic reviews included (Multimedia Appendix 2) were published between 2009 and 2016 in 16 journals. The systematic reviews involved 371 studies. After verifying the sample size of each study, we found that at least 79,665 participants were included. In these reviews, systematic literature searches were performed from 1950 to April 2015 (see Multimedia Appendix 2).

Of the reviews, 17 included studies that were performed in low- and middle-income countries, 13 included studies in multiple settings, 6 specified particular settings, and 2 did not describe the setting. mHealth modalities described were mainly apps for chronic diseases, but also for disease management, treatment adherence, and changes in health behavior. Nine studies performed a meta-analysis [13-21].

#### Objective

The main objective of the reviews was to analyze the impact or effectiveness of mHealth interventions on chronic and noncommunicable diseases. Other focuses were to analyze mHealth in supporting chronic diseases management, health behavior change, attendance at appointments, disease and rehabilitation management, and the use of mHealth strategies by health workers.

#### Intervention

Different devices were used, including mobile phones, smartphones, personal digital assistants, MP3, phone plus app, medical device connected to phone by cord or wirelessly, and many others.

The most frequent intervention was SMS for reminders, education, motivation, or prevention. Sensors and point-of-care diagnosis, data collection, provider-provider communication, patient-provider communication, decision support, client education, provider work planning, training, protocol-based treatment, voicemail, videos, immediate physician feedback from a central location, cloud-based interactive voice response, disease management calls, disease monitoring, automated email to clinicians, treatment adherence, and phone counseling were also used.

These interventions were performed for smoking cessation, to increase physical activity, chronic disease management, chemotherapy-related symptoms monitoring, sexual health behavior safety, alcohol consumption reduction, medication adherence, appointment attendance, stress management and anxiety reduction, vaccination timeliness, prenatal support, reduction in emergency referrals or adverse events, health information access, cardiopulmonary resuscitation skills, patient satisfaction, and social functioning.

Hall et al [22] categorized 12 common applications: (1) client education and behavior change, (2) sensors and point-of-care diagnostics, (3) registries and vital events tracking, (4) data collection and reporting, (5) electronic health records, (6) electronic decision support: information, protocols, algorithms, checklists, (7) provider-provider communication: user groups and consultation, (8) provider work planning, (9) provider training and education, (10) human resource management, (11) supply chain management, and (12) financial transactions and incentives.

Multiple interventions were used on significantly varied targets, and the duration of follow-up varied from a few minutes to up to 24 months.

#### Control Group

The control group care was not clear in some reviews [16,23,24], but others were very specific.

#### Outcomes

The primary outcomes assessed were clinical outcomes (eg, frequency of hypoglycemic events, symptoms, deaths), surrogate outcomes (eg, glycated hemoglobin [HbA_1c_], blood pressure, lipid profile, cardiovascular disease risk profiles, lung function tests results, nebulizer use, weight, body mass index [BMI]), behavioral or lifestyle changes (eg, sexual behavior, smoking cessation, increase in physical activity), and processes of care (eg, attendance rates, compliance with medication taking, data management, communication performance, time to diagnosis, time to treatment, changes in professionals’ workload).

The secondary outcomes were cost, patient satisfaction, and potential harms and adverse effects.

#### Quality of Included Studies

Multimedia Appendix 3 shows the results of the quality assessment of the 23 systematic reviews. Ten were rated low quality (AMSTAR score 0-4), seven moderate quality (score 5-8), and six were high quality (score 9-11).

Regarding bias risk assessment, 10 reviews did not explicitly report on study quality assessment. Two specified that the risk of bias was mostly either low or unclear [25]. Free et al [18,19] related that only 4 trials were at low risk of bias in all areas. All studies included in Car et al [13] had high risk of bias.

Baron et al used the McMaster University quality assessment tool and reported that the overall quality was poor [26]. Fanning et al used the Guide to Community Preventative Services data extraction form and reported that 7 studies were rated “fair” and 4 were “good” [16]. Three reviews [27] used the Cochrane Handbook for Systematic Reviews of Interventions [28] reporting varying methodological quality, with some providing insufficient information.

Two reviews reported adequate sequence generation for randomization [20]. De Jongh et al [17] reported adequate sequence generation in 3 of 4 and that none of the included studies were clear if the allocation was concealed. A potential for bias occurred from the apparent lack of blinding of outcome assessors. In Vodopivec-Jamsek et al [27], allocation concealment was considered adequate in 2 studies and unclear in 2 studies. Only one study reported on blinding of personnel collecting and analyzing samples. No mention is made of blinding of outcome assessors or researchers, which could have introduced bias.

Incomplete data analysis methods varied, with analysis and reporting based on intention-to-treat analysis and on only participants who completed the study, which could influence generalizability of the findings. Substantial heterogeneity was detected across analyses; however, a post hoc decision to conduct the main analysis using a random-effects model resulted in no difference in the interpretation of findings. Bacigalupo et al [29] also used Cochrane guidance [28], reporting that 4 of 7 studies presented low risk of bias. The only studies in which Grading of Recommendations, Assessment, Development, and Evaluation (GRADE) methodology [30] was used were those published in the Cochrane Database of Systematic Reviews [13,15,17,20,27].

### Main Results

The reviews show a variety of results, as expected, for using different devices and different mHealth interventions on different populations. The most widely used and successful intervention was SMS addressed to chronic disease patients. Positive impact was reported on clinical outcomes, adherence to treatment and care, health behavior changes, disease management, attendance rates, and others. However, some reviews showed conflicting results, with no significant differences between intervention and control groups.

#### Clinical Outcomes

##### Asthma

Positive impact has been demonstrated with moderate-quality evidence that text messaging interventions showed greater improvements in the pooled symptom score (mean difference 0.36, 95% CI -0.56 to -0.17) compared with the control group [17]. Very low-quality evidence (GRADE Very Low) showed the following: increased office visits in the SMS group, whereas increased hospital admissions for the control group [17]; a reduction in hospitalizations and better symptom control using home spirometry transmission to physicians via SMS and telephone counseling [25]; increased unscheduled visits to the emergency department and hospitalizations using mobile phone-based interactive self-care software plus management feedback on pulmonary function [25]; and improvements in cough and nighttime symptoms and decreased daily doses of medication using peak flow and symptoms monitoring via SMS [31].

##### Cardiac Rehabilitation

Exercise capacity in cardiac rehabilitation improved a 6-minute walk test from 524-637 meters (*P*=.009) in monitored exercise training assisted by a mobile phone app. In an 8-week, nonrandomized clinical trial, there was 17.6% (SD 16.1) improvement in mobile (n=30) versus 11.5% (SD 35.9) in control group (n=32) (*P*>.05) [32].

##### Congestive Heart Failure

Mobile technology counseling led to fewer symptom complaints in congestive heart failure subjects [25]. There was relative risk reduction (20%) of death or hospitalization and better quality of life with nurse telephone intervention and cardiologist support [33].

##### Chronic Lung Diseases

An SMS program improved cough symptoms and sleep quality [34]. Mobile phones recorded respiratory symptoms during exercise training that increased walking distance [34].

##### Chemotherapy Symptoms

No statistically significant effects were demonstrated on chemotherapy-related toxicity symptoms when patients used a mobile phone app to report symptoms and receive self-care advice [18].

##### Diabetes

Educational group sessions for diabetic women via SMS showed positive effects on sleep, positive actions, and coping [35].

#### Surrogate Outcomes

##### Asthma

There was moderate-quality evidence that text messaging intervention led to greater improvements in peak flow variability (mean difference -11.12, 95% CI -19.56 to -2.68) compared with the control group. No significant differences existed between groups in impact on forced vital capacity or forced expiratory flow in 1 second (GRADE Moderate) [17].

Forced expiratory flow in 1 second improved after 4 months of home spirometry transmission to physicians via SMS and telephone counseling [25]. Using mobile phone-based, interactive self-care software plus management feedback on pulmonary function showed evidence of improvement in pulmonary function and health-related quality of life and decreased unscheduled visits to emergency departments and hospitalizations, plus an increase in the proportion of participants who received leukotriene inhibitors [25].

##### Cardiac Rehabilitation

Improvement in at least 1 risk factor (relative risk [RR] 1.4, 95% CI 1.1-1.7) in a randomized controlled trial (RCT) that assessed the impact of lifestyle counseling, mobile intervention, devices for home monitoring plus SMS messaging of recommendations, compared to lifestyle counseling alone. The mHealth group was more likely to achieve goals for blood pressure (BP) (62.1% vs 42.9%), HbA_1c_ (86.4% vs 54.2%), and BMI (0.37 kg/m^2^decrease vs 0.38 increase). No significant differences in smoking cessation, cholesterol, or medication adherence [32].

##### Chronic Lung Diseases

Three RCTs showed nonsignificant results in lung function parameters [34].

##### Diabetes

Educational program via SMS for self-management improved HbA_1c_, low-density lipoprotein cholesterol, and microalbuminuria [33]. Cloud-based interactive voice response calls and automated email for clinicians reduced HbA_1c_ [33]. SMS promoting medication adherence reduced fasting plasma glucose and 2-hour, postprandial glucose [33]. SMS with tailored instructions on diabetes mellitus care to adolescents and elderly patients improved HbA_1c_ [34]. Diabetes self-management intervention reduced HbA_1c_ [18]. Diabetes education and advice via mobile phone and SMS significantly reduced HbA_1c_ [31]. A mobile phone-based, home glucose monitoring program study decreased HbA_1c_ from 13.2% to 10.5% after 3-6 months [35]. Text messaging improved HbA_1c_, with positive results in 6 of 8 studies [36]. Daily glucose readings were transmitted via mobile phone to a physician who made adjustments plus clinic appointment [25].

The results were mixed on the impact of mobile telemonitoring supporting diabetes management and feedback on HbA_1c_ but were more consistently positive for studies in type 2 diabetes. Ten of the 13 studies in type 2 diabetes and 4 of 7 studies on type 1 diabetes found mHealth led to HbA_1c_ improvement [26]. Studies without health care professional feedback led to HbA_1c_ improvement, suggesting professional feedback might not be necessary [26]

However, one study showed reduced benefit: educational group sessions for diabetic women via SMS showed higher diastolic blood pressure (+7 mmHg) and less spiritual hope at 6 months, and frequent texters had higher BMI and more sedentary time [35]

##### HIV Management

Text messages to maintain contact, monitor, and respond to medication issues in patients on antiretrovirals statistically significantly reduced human immunodeficiency virus (HIV) viral load by improving adherence [18].

##### Hypertension

Improvement in BP has been demonstrated with SMS or voice mail and immediate physician feedback [33]; SMS enabling interactive monitoring, where the provider sets reminders for patients, collects data, and schedules visits [34]; and electronic salt sensor and mobile sensor [34].

However, other reviews showed no benefit. Two trials showed no statistically significant reduction in BP [18]. Groups that did or did not receive alerts and reminders had nearly equal percentages of patients with controlled BP at follow-up [31].

##### Risk Factors for Coronary Artery Disease

Significant improvements were shown with automatic sphygmomanometer, blood glucose and lipid meter, and mobile phone [34].

##### Weight Loss

Moderate-quality evidence of short-term weight loss in overweight and obese adults with BMI of 25-39.9 using mHealth structured program was shown [29]. Mobile phone personalized advice and motivation observed a significant improvement in percent of body fat lost; however, BMI and systolic and diastolic BP were unchanged [31].

#### Behavioral or Lifestyle Changes

##### Physical Activity

Seven of 14 trials reported statistically significant benefits on self-reported physical activity outcomes, but no statistically significant change was demonstrated on trials using SMS to reduce calorie intake and increase physical activity or for trials targeting physical activity only, diet only, or diet and physical activity [18].

##### Smoking Cessation

Positive results have been demonstrated with moderate-quality evidence that mobile phone-based cessation interventions increased abstinence rates at 26 weeks (RR 1.67 95% CI 1.46-1.90, 12 RCTs in high income countries, 11,885 participants, GRADE Moderate). Six studies verified quitting biochemically at 6 months (RR 1.83 95% CI 1.54-2.19) [20]. SMS-based smoking cessation interventions doubled biochemically verified smoking cessation at 6 months [18].

##### Sexual Behavior

One study showed statistically significant benefits on behavior change [18].

#### Processes of Care

##### Antenatal Support

Pregnant women connecting to their health care provider through bidirectional mobile phone messaging were more likely to have skilled birth attendants [37].

##### Attendance Rates

A consistent improvement on attendance rates has been demonstrated. Text message reminders improved the rate of attendance at health care appointments compared with no reminders (RR 1.10; 95% CI 1.03-1.17) (7 studies, 5841 participants, GRADE Moderate) [15]. They had a similar impact to phone call reminders (RR 0.99, 95% CI 0.95-1.02) (3 studies, 2509 participants, GRADE Moderate). Text messages plus postal reminders improved attendance rates at appointments compared to postal reminders (RR 1.10, 95% CI 1.02-1.19) (1 study, 291 participants, GRADE Low) [13].

In a limited study, Shetty et al [38] compared the effect of one SMS reminder sent to type 2 diabetes patients every third day for 1 year. Although there was no significant reduction in the mean HbA_1c_ values in either group, the percentage of patients with HbA_1c_<8% decreased significantly in the SMS group. Free et al [18] included one study assessing SMS reminders on attendance for vaccination at different time points (as different studies). The relative risk was 1.19 (95% CI 1.15-1.23), but there was significant heterogeneity (I^2^=99.7%, *P*<.001) [33]. In 8 studies Free et al [19] observed that the pooled effect on appointment attendance using text message reminders versus no reminder was RR 1.06 (95% CI 1.05-1.07) [19]. There was no effect on the number of cancelled appointments (RR 1.08, 95% CI 0.89-1.30), and no difference in attendance using SMS reminders versus other reminders (RR 0.98, 95% CI 0.94-1.02) [19].

Hall et al [22] assessed 6 studies in low- and middle-income countries. The studies showed mHealth interventions to be beneficial, except a pilot study in rural Swaziland [39]. Mahmud et al [40] trained community health workers to use mobile phones for reporting on patient adherence, send appointments reminders, and answer physician queries. This evaluation was based on a retrospective observational study, with the possibility of recall bias.

##### Data Collection and Health Care Team Communication

Studies that included data collection as a primary mHealth function demonstrated that mobile phones effectively collect and report data, transfer patient-relevant information, and reduce the need for face-to-face communication [37]. There is a reduction in communication delays and improvement on data collection and reporting [23]. One trial reported a statistically significant improvement in nurse/surgeon communication using mobile phones [19].

##### Adherence to Treatment

mHealth strategies are beneficial to increase adherence to treatment in diabetes patients: SMS to increase adherence to prescriptions in type 2 diabetes [25], electronic blister packs with SMS communication [34], and insulin adherence among type 1 diabetes patients who received tailored text messages with goal-specific prompts [31].

Compliance with medication taking among memory-impaired, HIV-positive patients significantly increased compared to those without impairment. Hepatitis A and B dose vaccination schedules also increased among international travelers with reminders sent to mobile phones [31].

Hall et al reported improved adherence to tuberculosis treatment by using text messages and adherence to HIV therapy, with evidence of reduction of viral load [22], although the authors did not perform quality assessment. The authors commented that a risk-benefit analysis that assessed mHealth reminders to improve tuberculosis medication adherence showed increased mortality and disability-adjusted life years compared to directly observed treatments, and that an RCT in China observed no benefit with voice calls. They also reported limited evidence of contraceptive knowledge improvement with the use of an SMS education scheme; risk reduction of contracting dengue fever, but no significant improvement over alternative schemes; antenatal care improvement, with increases in using skilled birth attendants and women with 4 antenatal visits; and improved vaccination rates in rural Kenya [22].

Hamine et al observed in 2 studies that text messaging tailored to counteract negative beliefs about asthma and education was associated with improved adherence to medication [34].

##### Diagnosis

Hall et al mentioned studies showing improvement of diagnostic rates of dermatological conditions with mobile teledermatology [17]. However, two trials using mobile phones to transmit photos to offsite clinicians reported significant reductions in correct diagnoses compared to an onsite specialist [18]. One trial reported reduction in quality of electrocardiography (ECG) transmitted via mobile phone to an ECG transmitted by fax, but with no effects on ECG interpretation [18]. Krishna et al [31] showed that fewer days to diagnosis and treatment were reported among those who were notified of test results via text messages.

#### Cost

The following studies assessed costs, with evidence of reduction when compared to control groups. SMS reminders were more cost-effective than telephone and were equally efficacious [25]. SMS was found to be 35% and 45% less expensive, respectively, per attendance through reductions in research assistants’ work hours and in telecommunications costs. SMS reminders were less expensive than mobile phone reminders [33,40]. The relative cost of the text message per attendance was 55% and 65% of the cost of phone call reminders [13]. There was a reduction in patient burden to transportation time and costs in African countries [23]. The cost of text-based support per 1000 enrolled smokers was GB 278 per quitter, but when future health service costs were included, text-based support was considered a cost-saver [20].

#### Patient Satisfaction

The individual studies did not assess this outcome.

#### Potential Harms and Adverse Effects

Only 3 reviews assessed this outcome [15,17,27]. Vodopivec-Jamsek et al [27] reported one study where mobile phone messaging was used to support smoking cessation and that messaging did not have any impact on rates of pain in the thumb or finger joints (RR 1.08, 95% CI 0.74-1.59), or car crash rates (RR 0.88, 95% CI 0.58-1.35) at 26 weeks follow-up.

## Discussion

### Principal Findings

The current evidence shows benefits of mHealth in chronic disease management, improving symptoms and peak flow variability in asthma patients and chronic pulmonary diseases symptoms; heart failure symptoms, reducing deaths and hospitalization and improving quality of life; glycemic control in diabetes patients; and improving BP in hypertensive patients. SMS reminders improved attendance rates at reduced costs and improved adherence to tuberculosis and HIV therapy in some scenarios, with evidence of decrease of viral load.

Mobile devices may improve patient-provider communication, facilitating assistance in disease management. It may increase the likelihood of delivering health interventions to hard-to-reach populations. Whittaker et al [20] listed advantages of using mobile interventions: convenience, ease, cost-effectiveness, scalability, personalization, and “the ability to send time-sensitive messages with an ‘always on’ device.” Hamine et al [34] observed that mHealth tools can impact patients who are less inclined to engage with traditional health services. Aranda-Jan et al [23] reported that governments may benefit from increased support of patient management and increased direct communication in rural areas. Health workers may receive support through professional networks and can prioritize efforts and increase their role in active case detection using disease surveillance systems. Baron et al [26] assessed studies involving data transferring for diabetes management and suggested that recording and tracking of data might increase patients’ motivation to self-manage.

The most popular mHealth intervention was behavior change interventions using text messaging. The low cost and low broadband requirements facilitate the spread of applications, even in low-income countries.

Different uses of motivation have also been described as a tool to be used in mHealth interventions in some of the systematic reviews analyzed. These are mainly focused on patient motivation in different contexts on chronic diseases [36], communicable diseases [23], physical activity [16,29,31], and empowerment in the use of services [37].

Two reviews [14,32] reported lessons learned: patient needs must be met, training and support provided, users engaged in the development and implementation of the tools, and consideration of patient age and education level. Usage might improve with user-centered design, engagement strategies, and feedback to the users.

This study provides a thorough review of available evidence on effectiveness of mHealth interventions in different health conditions and in the processes of health care service delivery, so it useful to guide clinical and health policy decisions. However, there are some limitations of the studies that need to be addressed.

Studies assessing mHealth interventions usually do not include the assessment of risks, consumer satisfaction, and acceptability of the intervention [17]. None reported studies assessing security and confidentiality. Chen et al [41] noted that mobile phone numbers frequently change in China, reducing certainty the message was delivered to the correct recipient. This was not assessed elsewhere. Particularly in low-income countries where mobile phones are frequently shared, these are important confidentiality issues that must be taken into account when designing interventions [15]. De Jongh et al [23] warned of inaccurate data input, misinterpretation of the information, and difficulties in reading due to vision or literacy problems and remarked that text messaging cannot capture verbal and nonverbal cues. Norwell suggested that doctors agree on vocabulary to minimize the risk of patients’ misunderstanding the message [38]. Risks associated with mobile phone messaging in general may apply, such as car accidents [23].

Other drawbacks related to mHealth initiatives were reported by Hamine et al [34] Some patients’ concerns included dependence on professional supervision, unnecessary medicalization, fear of technology failure, and difficulty in understanding and using the technology. Provider concerns related to data review and response times, increased clinical workload and workflow, record maintenance, and concerns about supervision and technology dependence. Aranda-Jan et al [23] reported difficulties in monitoring text message content, data underreporting, and the possibility of biased responses from participants.

Two reviews cite availability and poor connectivity as barriers [34]. Most identified the main limitation as the small number of RCT studies, patients enrolled, and the low-to-moderate quality of evidence. Researchers should validate their pilot study findings through follow-up studies with adequate research designs and appropriate controls [17]. Aranda-Jan et al [23] mention that claimed benefits are unclear and long-term results uncertain. Also, only 2 reviews assessed funding [15]. This is important to identifying conflicts of interest. To improve the suboptimal reporting and standardize Web-based and mobile health interventions, the CONSORT-EHEALTH was developed, a checklist that is an extension of the CONSORT statement [42].

Costs have not been routinely assessed. Such costs may be dependent on the nature of the intervention and the size and characteristics of the target group [17]. More attention to cost implications seems warranted [27]. Additionally, future studies should compare effects in different contexts [27].

### Conclusion

Although mHealth is growing in popularity, the evidence for efficacy is still limited. Positive results were reported for chronic disease management, improving chronic pulmonary diseases symptoms and heart failure symptoms, reducing deaths and hospitalization and improving quality of life, and improving glycemic control in diabetes patients and BP in hypertensive patients. SMS reminders improved attendance rates and improved adherence to tuberculosis and HIV therapy in some scenarios. However, in general the methodological quality of the studies included in the systematic reviews is low. For some fields, its impact is not evident or is mixed. Exceptions are the moderate improvement in asthma patients, attendance rates, and smoking cessation rates.

## Appendix

Multimedia Appendix 1 Search methods and strategy.

Multimedia Appendix 2 Descriptive summary of the 23 systematic reviews included in mHealth review.

Multimedia Appendix 3 Quality assessment ratings of systematic reviews included in mHealth review.

## Abbreviations

AMSTAR: Measurement Tool to Assess Systematic Reviews
BP: blood pressure
BMI: body mass index
CONSORT: Consolidated Standards of Reporting Trials
ECG: electrocardiography
GRADE: Grading of Recommendations, Assessment, Development and Evaluation
HbA_1c_: glycated hemoglobin
IBECS: Indice Bibliográfico Español de Ciencias de la Salud
LILACS: Literatura Latino-Americana e do Caribe em Ciências da Saúde
mHealth: mobile health
PRISMA: Preferred Reporting Items for Systematic Reviews and Meta-Analyses
RCT: randomized controlled trial
RR: relative risk
SD: standard deviation
SMS: short message service
