# Supplementary material for: The Impact of mHealth Interventions: Systematic Review of Systematic Reviews
Source: JMIR Mhealth Uhealth. 2018 Jan 17;6(1):e23. doi: 10.2196/mhealth.8873 (PMC5792697; doi:10.2196/mhealth.8873)
Supplement: Multimedia Appendix 1 [file mhealth_v6i1e23_app1.pdf]

Supplementary file 1. Search methods and strategy

On Pubmed, the search strategy was: ("Telemedicine"[Mesh] OR "Remote Consultation"[Mesh] OR telehealthcare OR Telemonitoring OR “remote monitoring” OR Tele-education OR “health messages” OR “educational technology” OR “decision support system” OR “remote diagnosis” OR telediagnosis OR videoconferencing OR telemanagement OR tele-management OR teleconsult*) AND ("Cost-Benefit Analysis"[Mesh] OR "Evaluation Studies"[Publication Type] OR "Program Evaluation"[Mesh] OR impact or effectiveness).

On IEEE, as there was a limitation of maximum 15 terms for the search, seven searches were performed:

1. (telemedicine OR “mobile health” OR mHealth) AND ((analys* AND cost-benefit) OR cost OR effectiveness OR cost-utility OR (economic AND evaluation) OR (program AND (evaluation OR sustainability)) OR impact OR “clinical trial”)

2. (telehealth OR eHealth OR “remote consultation”) AND ((analys* AND cost-Benefit) OR Cost OR effectiveness OR cost-utility OR (economic AND evaluation) OR (program AND (evaluation OR sustainability)) OR impact OR “clinical trial”)

3. (teleconsult* OR telehealthcare OR telemonitoring) AND ((analys* AND cost-Benefit) OR Cost OR effectiveness OR cost-utility OR (economic AND evaluation) OR (program AND (evaluation OR sustainability)) OR impact OR “clinical trial”)

4. (“remote monitoring” OR tele-education OR “health messages”) AND ((analys* AND cost-benefit) OR cost OR effectiveness OR cost-utility OR (economic AND evaluation) OR (program AND (evaluation OR sustainability)) OR impact OR “clinical trial”)

5. (“educational technology” OR “decision support system” OR “remote diagnosis”) AND ((analys* AND cost-Benefit) OR cost OR effectiveness OR cost-utility OR (economic AND evaluation) OR (program AND (evaluation OR sustainability)) OR impact OR “clinical trial”)

6. (telediagnosis OR videoconferencing OR telemanagement) AND ((analys* AND cost-Benefit) OR cost OR effectiveness OR cost-utility OR (economic AND evaluation) OR (program AND (evaluation OR sustainability)) OR impact OR “clinical trial”)

7. (tele-management OR teleconsult) AND ((Analys* AND Cost-Benefit) OR Cost OR effectiveness OR Cost-Utility OR (economic AND evaluation) OR (program AND (evaluation OR sustainability)) OR impact OR “clinical trial”)

On Virtual Health Library, the search strategy was: (telemedicine OR “mobile health” OR mHealth OR telehealth OR eHealth OR “remote consultation” OR teleconsult* OR telehealthcare OR telemonitoring OR “remote monitoring” OR tele-education OR “health messages” OR “educational technology” OR “decision support system” OR “remote diagnosis” OR telediagnosis OR videoconferencing OR telemanagement OR tele-management OR teleconsult) AND ((analys* AND cost-Benefit) OR cost OR effectiveness OR cost-utility OR (economic AND evaluation) OR (program AND evaluation) OR impact OR effectiveness OR “clinical trial” OR (program AND sustainability)).
