# Supplementary material for: The Impact of mHealth Interventions: Systematic Review of Systematic Reviews
Source: JMIR Mhealth Uhealth. 2018 Jan 17;6(1):e23. doi: 10.2196/mhealth.8873 (PMC5792697; doi:10.2196/mhealth.8873)
Supplement: Multimedia Appendix 2 [file mhealth_v6i1e23_app2.pdf]

# Supplementary file 2. Descriptive summary of the 23 systematic reviews included in mHealth review

| First author | Year | Reference | Journal | Scenario/ setting | Theme/Speciality | Countries | Databases | Number of included studies | Study design of included studies | Meta-análise | Patients: total (n) | Mean age of included patients (years) | Male sex (%) |
|---|---|---|---|---|---|---|---|---|---|---|---|---|---|
| Beratarrechea | 2014 | [1] | Telemedicine and eHeallth | Multiple | mHealth on chronic diseases in developing countries | China, Taiwan, Malaysia, Poland, India, Croatia and Uruguay | CENTRAL, MEDLINE, EMBASE and LILACS, also grey literature and reference lists of included articles | 9 | RCT | N | 4604 | Not reported | Not reported |
| Peiris | 2014 | [2] | Journal of Cardiovascular Translational Research | Urban and rural settings | mHealth for non-communicable diseases in low and middle income countries | Brazil, Turkey, China, Honduras, India, Malaysia, Cameroon, Thailand, Iran, Argentina, Mexico | Medline, PsycINFO, EMBASE, CINAHL, Cochrane and LILACS, also grey literature and trial registries | 24 | RCT (7), quasi-experimental studies (6), descriptive studies (6), reviews (5) | N | Not reported | Not reported | Not reported |
| Hamine | 2015 | [3] | Journal of Medical Internet Research | Multiple | mHeallth on chronic disease management and treatment adherence | USA, UK, South Korea, India, China, Africa | Medline, EMBASE, CINAHL, PsychInfo and PsychArticles) | 107 | RCT (50); descriptive (26); longitudinal/pre-pos (13); quasi-experimental (10); crossover (7); retrospective (1 | N | Not reported | Not reported | Not reported |
| Free (a) | 2013 | [4] | PLOS Medicine | Multiple | mHealth on health behavior change and disease management | Health Behavior change: all studies in multiple high-income countries. Disease management: low-income countries (1), middle-income countries (2), all other in high-income countries. | MEDLINE, EMBASE, PsycINFO, Global Health, Cochrane Library, NHS Health Technology Assessment Database and Web of Science, also reference lists of included articles | 75 (49 on disease management and 26 in change health behaviors) | Health behavior: RCT (26). Disease management: RCT (34), cluster RCT (3), crossover (5), non-randomized parallel group trial (7) | Y | Health behavior change: 10706 Disease management: 6832 | Not reported | Not reported |
| Agarwal | 2015 | [5] | Tropical Medicine and International Health | Primary care | mHealth use by frontline health workers in developing countries | Most studies were located in Africa and South Asia. Few studies in South America | MEDLINE, EMBASE, Global Health, Scopus and Google Scholar | 42 | No reference | N | Not reported | Not reported | Not reported |
| Krishna | 2009 | [6] | Telemedicine and eHeallth | Multiple | mHealth on health behavior change, clinical improvement and social functioning | Australia, UK, Korea, New Zealand, Spain, US, Austria, China, Croatia, Italy, France, Netherlands and Norway | MEDLINE | 25 | RCT (20); CT (5) | N | 38060 (10374 adults and 27686 children). | Not reported | Not reported |
| Bloomfield | 2014 | [7] | Globalization and Health | Multiple | mHealth for non-communicable diseases in low and middle income countries | Sub-Saharan Africa (Cameroon, Kenya, Zambia, Nigeria, South Africa) | PubMed, EMBASE, CENTRAL, Web of Science, CINAHL, IEEE Xplore, PsycINFO, Global Index Medicus, Association for Computing Machinery Digital Library, Science Direct and Pan African Clinical Trials Registry, also grey literature reference lists of included articles | 5 | Not reported | N | Not reported | Not reported | Not reported |
| Car | 2012 | [8] | The Cochrane Database of Systematic Reviews | Multiple | mHealth on health behavior change | China, Scotland, England and Malaysia | CENTRAL, MEDLINE, EMBASE, PsycINFO, CINAHL, LILACS and African Health Anthology, also grey literature and reference lists of included articles | 4 | RCT (4) | Y | 3547 | 33, 38, 57. The forth study did not provide age information | Proportion of males ranged from 35 to 58% (three studies). The forth study did not provide gender distribution |

| First author | Year | Reference | Journal | Scenario/ setting | Theme/Speciality | Countries | Databases | Number of included studies | Study design of included studies | Meta-análise | Patients: total (n) | Mean age of included patients (years) | Male sex (%) |
|---|---|---|---|---|---|---|---|---|---|---|---|---|---|
| Free (b) | 2013 | [9] | PLOS Medicine | Multiple | mHealth on health behavior change and disease management | Health care provider support: multiple in high-income countries Communication between health care services and health care consumers: high-income countries (4) and in middle-income countries (3) | MEDLINE, EMBASE, PsycINFO, Global Health, Cochrane Library, NHS Health Technology Assessment Database and Web of Science, also reference lists of included articles | 42 (Heath care provider support: 32; Communication between health care services and health care consumers: 10) | Heath care provider support: RCT with parallel groups (15), crossover (6), cluster RCT (3), non-randomized controlled trial (8) Communication between health care services and health care consumers: RCT with parallel groups (7), non-randomized controlled parallel groups trial (3) | Y | Heath care provider support: 5323 Communication between health care services and health care consumers: 4473 | Not reported | No reference |
| Valles-Ortiz | 2015 | [10] | Enfermaría GlobaL | Doctor's office, Health Care Center or Clinic for Diabetes | mHeallth on chronic disease management: glicemic control in adult type 2 diabetes patients | South Korea (n=1), USA (n=2), India (n=1), Iran (n=2), United Kingdon (n=2) | Cochrane, CINAHL, DARE, Academic Search Complete, MedicLatina, Open Acess, MEDLINE, Cochrane Central Register of Controlled Trials, ScienceDirect and Academic Google | 8 | RCT (5), CT (2), and pre-test/post-test studies (2) | N | 812 | Mean age varied from 47 to 58 years-old | Varied from 23 to 78% |
| Hall | 2014 | [11] | Glob Health Action | Multiple | mHealth for health promotion and education, diagnosis, treatment/vaccination compliance, data collection and reporting, health records, case detection of communicable diseases, management of malnutrition, provider training and education | Low and middle-income countries (Tailand, South Africa, Argentina, Kenya, China , Benin, Botswana, Egypt, Malawi, Nigeria, Rwanda, Swaziland, Tanzania, Uganda, Brazil, Mexico, Peru, Cambodia, Indonesia, India, Pakistan, Bangladesh, Kosovo) | Medline and Google Scholar | 76 | No restriction | N | Not reported | Not applicable | Not reported |
| Aranda-Jan | 2014 | [12] | BMC Public Health | Multiple | Experiences of mHealth implementation in Africa. | African countries | Medline and OvidSP | 44 | No restriction | N | Not reported | Not reported | Not reported |
| Beatty | 2013 | [13] | Journal of the American Heart Association | Outpatients | mHealth for cardiac rehabilitation | Australia (n=1), Poland (n=1), Spain (n=1) | Medline | 3 | Observational (1), nonrandomized CT (1), RCT (1) | N | Not reported | Not reported | Not reported |
| Baron | 2012 | [14] | Journal of Diabetes Science and Technology | Not reported | mHeallth on chronic disease management: glicemic control in adult type 1 (n=7) and 2 (n=13) diabetes patients | Asia (n=8), Europe (n=8), USA (n=3) and one multinational trial | HMIC, Amed, Cochrane, Psycinfo, Embase, Medline | 20 | RCT (n=11), single group pre and post (5), crossover (2), controlled trial (pre and post)(1), cluster RCT (1) | N | 1840 | Mean age varied from 23.8 to 63.9 years-old | Varied from 28.1 tto 80.0% |
| Guy | 2012 | [15] | Health Research and Educational Trust | Primary care clinics, hospital outpatient clinics | mHealth for reminders for attendance at healthcare appointments | UK (n=4), Australia (n=3), Scotland (n=2), Malaysia (n=2), Ireland (n=1), USA (n=1), Denmark (n=1), Brazil (n=1), Korea (n=1), Netherlands (n=1), China (n=1) | Medline, Embase, Cochrane Controlled Trials Register and google. References lists were checked. | 18 | RCT (8), observational studies with concurrent controls (5) and observational with historical controls(5) | Y | 123111 (the unit is the appointment and not the patient) | Not reported | Not reported |

| First author | Year | Reference | Journal | Scenario/ setting | Theme/Speciality | Countries | Databases | Number of included studies | Study design of included studies | Meta-análise | Patients: total (n) | Mean age of included patients (years) | Male sex (%) |
|---|---|---|---|---|---|---|---|---|---|---|---|---|---|
| Jongh | 2012 | [16] | The Cochrane Database of Systematic Reviews | Multiple | mHealth for self-management of long-term illnesses | Scotland, USA, Spain and Croatia | The Cochrane Central Register of Controlled Trials, Medline, Embase, PsyInfo, CINAHL, Lilacs, African Health Anthology, grey literature (inluding trial registers) and reference lists | 4 | RCT with at least 3 time points before and after que intervention | Y (only for glycemic control) | 182 | Not reported.You th and youg adults for studies in diabetes patients, age over 18 years in the study in hypertensive patients and 24.6±6.5 years in the study in asthma patients | Not reported. It is mentioned that all studies included men and women in approximatel y equal ratios |
| Fanning | 2012 | [17] | Journal of Medical Internet Research | Not reported | mHealth for physical activity behavior | Not reported | PubMed, PsychINFO, SCOPUS | 11 | Not reported | Y | 1351 | Mean age varied from 8.7 to 68 years | Not reported |
| Gurol-Urganci | 2012 | [18] | The Cochrane Database of Systematic Reviews | Multiple | mHealth for reminders for attendance at healthcare appointments | Australia (n=1), China (n=2), Scotland (n=1), England (n=1), Kenya (n=1), Malasya (n=2) | The Cochrane Central Register of Controlled Trials, Medline, Embase, PsyInfo, CINAHL | 8 | RCT. The unit of randomization was individual participants (7) or the healthcare appointment (1) | Y | 6615 | Mean age varied from 29 to 59 years | The proportion varied from 35 to 65% in 6 studies, one study included only men and one did not provide information |
| Vodopivec-Jamsek | 2012 | [19] | The Cochrane Database of Systematic Reviews | Multiple | mHealth for preventive health care | Canada (n=1), Thailand (n=1), New Zeland (n=1) and USA (n=1) | The Cochrane Central Register of Controlled Trials, Medline, Embase, PsyInfo, CINAHL, Lilacs, African Health Anthology, grey literature (inluding trial registers) and reference lists | 4 | RCT with at least 3 time points before and after que intervention | N | 1933 | Varied from 23.8-27.3 years-old in 3 studies. One study included only children. | Varied from 38-45%, and one study included only women (antenatal care) |
| Fjeldsoe | 2009 | [20] | American Journal of Preventive Medicine | Multiple | mHealth for health behaviour change | New Zeland (n=1), USA (n=1), UK (n=2), Korea (n=2), Finland (n=1), Scotland (n=1), Austria (n=2), South Korea (n=1), Croatia (n=1), Spain (n=1), Canada/USA (n=1) | Medline, Pubmed, ERIC, Web of Science and PsyINFO | 14 | RCT (6), cluster RCT (1), randomized crossover trial (1), single group pre-post desing studies (6) | N | 3512 | Not reported | Not reported |
| Whittaker | 2016 | [21] | The Cochrane Database of Systematic Reviews | Outpatients | mHealth for smoking cessation | USA (n=3), Australia (n=2), UK (n=34, Switzerland (n=1), New Zeland (n=2) | The Cochrane Central Register of Controlled Trials, Medline, Embase, PsyInfo, UK Clinical Research Network Portfolio, US ClinicalTrials.gov and reference lists | 12 | RCT | Y | 11885 | Not reported | Not reported |
| Liang | 2011 | [22] | Diabetic Medicine | Multiple | mHeallth on chronic disease management | Not reported | PubMed, EMBASE, Cochrane Library | 22 | RCT (11), quasi-randomized trials (2), randomized crossover trials (2), controlled before–after trial (7). | Y | 1657 | Not reported | Not reported |

| First author | Year | Reference | Journal | Scenario/ setting | Theme/Speciality | Countries | Databases | Number of included studies | Study design of included studies | Meta-análise | Patients: total (n) | Mean age of included patients (years) | Male sex (%) |
|---|---|---|---|---|---|---|---|---|---|---|---|---|---|
| Bacigalupo | 2013 | [23] | Obesity Reviews | Outpatients | mHeath for for physical activity and diet behavior | USA (n=3), Germany (n=2), Finland (n=1), UK (n=1), | Medline, Embase, Science Citation Index, Social Science Citation Index, Cumulative Index to Nursing and Allied Health Literature, the Cochrane Central Register of Controlled Trials, Meta register of controlled trials, ClinicalTrials.gov and reference lists | 7 | RCT | N | 584 | Varied from 25 to 70 years-old | Varied from 20 to 95% |

## Supplementary file 2. Continued

| First author | Year | Reference | Intervention | Target of intervention | Duration of follow up (months) | Outcomes |
|---|---|---|---|---|---|---|
| Beratarrechea | 2014 | [1] | Voice communication and short message service | Patients | 4 months to 1 year, | Clinical outcomes, processes of care, costs, patient-provider compliance, and HRQoL |
| Peiris | 2014 | [2] | The mHealth interventions were characterized using a framework (Labrique *et al.*): client education and behavior communication, sensors and diagnostic, registries, data collection, electronic health records, decision support, provider communication, provider work-planning and supply chain management. Most studies tested only 1 or 2 mHealth interventions. | Patients, volunteers | 5, 6 week; 3 to 24 month | Quality of care |
| Hamine | 2015 | [3] | The use of devices that allowed patients to use short message service, medication reminders, symptom monitoring, educational tools and facilitated patient-provided communication. The focus were vulnerable, hard-to-reach, high-risk patient populations (elderly patients, minority ethnic and racial groups and low-income adults). Chronic diseases: diabetes, cardiovascular disease (hypertension, coronary artery diseases, congestive heart failure) and chronic lung disease. | Patient and provider (to acceptability) | Few hours to 18 months | mAdherence to chronic diseases management |
| Free (a) | 2013 | [4] | Health behavior change: of the 26 studies, 16 used mobile phones, of which MS function was used by 13, MP3 function by 1, MP4 by 1, and the telephone function by 1. Six studies used PDA phone with application software, SMS and telephone functions. Two studies used hand held computers employing application software or MP4/video functions. One used MP3 audio players. Disease management: of the total of 49 interventions, 42 used mobile phone or PDA, with SMS (27), MP4 video (3), MP3 audio and MP4 video (4), application software (6), WAP for data transfer (1), and telephone functions (1). Two interventions were delivered via PDA, three used hand held computers, and one used a video console all of which employed application software. One used a portable media player/MP3. | Multiple | Few minutes, few hours, 9 days, 4 to 14 weeks; 3 to 12 months | Primary and secondary outcomes for health behavior change and disease management |

| First author | Year | Reference | Intervention | Target of intervention | Duration of follow up (months) | Outcomes |
|---|---|---|---|---|---|---|
| Agarwal | 2015 | [5] | Training in the use of mobile phones. Use of mobile phones for decision support tools, emergency referrals, supervision, alerts and reminders, client education, data collection and reporting. | Frontline health workers | Not reported | Effectiveness and feasibility of use of mobile tools, training required for adoption of mobile tools. |
| Krishna | 2009 | [6] | The technology used in all 25 studies was voice or the SMS feature of cell phones. Most studies used “Push” technology where participants received personalized text messages or automated voice mail messages delivered to their specific health needs and personal preferences. Frequency of message delivery ranged from daily to once a week and varied by disease or behavior modification area | Patients | 3 weeks to 12 months | Process of care (activities involved in the delivery of healthcare): notification of diagnoses, recall of patients with positive lab results to the clinic for treatment consultation, appointment reminder, and teach persons with disabilities to improve communication. Outcomes of care (change in disease-specific health outcomes): behavior change including smoking cessation, compliance with medication taking, and getting timely vaccinations; clinical improvement including diabetes control and management (reduction in HbA1c), asthma (peak flow and symptoms monitoring), hypertension, stress management (State-Trait Anxiety Inventory score), and physical activity (body fat lost, body mass index, and systolic and diastolic blood pressure); and social functioning including quality of life, satisfaction with life and self-efficacy |
| Bloomfield | 2014 | [7] | (1) use of mobile phones by guardians of pediatric patients with Burkitt’s lymphoma in two hospitals in Northwest Cameroon. (2) a mobile phone-based home glucose monitoring program was created in which community health workers provided clinical consultation for diabetic patients via mobile phone. (3) a system operated by non-physician health providers in a public sector cervical cancer prevention program. After taking photographs of a suspicious cervical lesion with a mobile phone, nurses in remote settings sent images electronically to an expert consultant for review as well as an SMS message notifying the consultant to review the images. (4) a Nigerian teaching hospital gave their oncologists’ mobile phone numbers and advised to call to seek medical advice at any time. (5) a series of educational group sessions addressing lifestyle improvements were offered to diabetic women and each was assigned a “text message buddy” to assist with lifestyle changes via SMS. | Patients and professionals | Not reported | Primary outcome measures included behavioral and clinical measures depending on the study design. (1) Access to mobile phones and successful contact, (2) change in HbA1C with home glucose monitoring, (3) feasibility of digital cervicography intervention, (4) call reason and duration, patient perception, and clinic attendance, (5) uptake of text messaging, body mass index, blood pressure, styles of coping, emotional distress, and sedentary time. |
| Car | 2012 | [8] | The purpose of mobile phone messaging was to remind the participant of their upcoming healthcare appointment. Different plataforms were used including a web-based provider and via Global System for Mobile (GSM) modem linked to an eletronic health records system. The timing of the reminder varied from 24, 48 and 72 hours before the appointment. | Patients and professionals | 2, 3, 7 and 9 months | Primary outcome: rate of attendance at healthcare apoointments. Secondary outcomes: health outcomes as a result of the intervention, including physiological measures, e.g. blood pressure; clinical assessments; biomarker values; self reporting of symptom resolution or quality of life; user evaluation of the intervention, including satisfaction, readiness to use, timeliness, availability and/or convenience; user perceptions of safety; costs; potential harms or adverse effects of the intervention, such as misreading or misinterpretation of data, transmission of innacurate data, loss of verbal and non-verbal communication cues, issues of privacy and disclosure, or failure or delay in the message delivery |

| First author | Year | Reference | Intervention | Target of intervention | Duration of follow up (months) | Outcomes |
|---|---|---|---|---|---|---|
| Free (b) | 2013 | [9] | Health care provider support: seven investigated interventions providing health-care provider education (six used application software delivered via personal digital assistants and one employed a MP4/video technology using a portable media player), 18 investigated interventions supporting clinical diagnosis and treatment (14 trials used customised application software being 12 on personal digital assistants, one on a tablet PC, one on a handheld computer, and four used photographs and video capabilities using mobile phones), and seven investigated interventions to facilitate communication between health-care providers (three trials relied on the use of MMS for sending images by mobile phone, one used the telephone function of the mobile phone, one used MMS on a PDA, one made use of MP4/video technology and the other made use of installed customised software, using hand-held computers).<br>Communication between health services and consumers: All the interventions used SMS messages delivered by mobile phone. One appointment reminder trial also used voice messages. Eight of these trials investigated SMS-based appointment reminders and two investigated test result notification. | Patients and professionals | Not reported | Health care provider support: for medical education interventions, one trial reported two outcomes regarding documentation of health care problems and four trials reported nine knowledge outcomes. For clinical diagnosis and management interventions, seven trials reported 25 outcomes relating to appropriate management (3 outcomes), testing (3), referrals (1), screening (4), diagnosis (2), treatment (2), and triage (10). Six trials reported 17 medical process outcomes: perceived difficulty in performing a task (1 outcome), use of tool (1), errors in report (2), errors in score calculation (2), completeness of reports (2), time to complete a report (2), time to record vital signs (1), time to diagnosis (3), and time to treatment (3). For interventions using mobile technologies to communicate between health care providers for clinical/patient management outcomes, six trials reported 19 outcomes relating to the quality of nurse surgeon communication (6 outcomes), correct clinical assessment or diagnosis (4), test score (1) and electrocardiogram (ECG) transmission (8), feasibility of delivery (1), time taken (4), and quality (3).<br>Communication between health services and consumers: eight trials reported appointment attendance and two trials reported cancelled appointments as an outcome. For patient notification of test results, outcomes were time to diagnosis (1), time from first contact to treatment (1) and time from test to treatment (1), and anxiety scores (2). |
| Valles-Ortiz | 2015 | [10] | The use of text messages sent through mobile phone service to adults involved in self-management of type 2 diabetes to maintain their glycemic control and report on their HbA1c, also involving educational and motivational messages in 5 studies. Six of the 8 studies analyzed maintained contact exclusively through text messages. In one study, one weekly phone call was made by the patient's health provider if he had not received information about the patient three days after the date appointed to communicate results while another study compared the use of SMS with phone calls made to land lines. Intervention studies which used the sending of text messages with information concerning self-care, management of prescribed therapeutics, clarification of doubts and survillance of blood glucose were excluded. | Patients | 3 - 12 months | Hb1Ac improvement |
| Hall | 2014 | [11] | Health impacts of mHealth interventions, categorised by 12 common applications of mHealth: Client Education and Behaviour Change; Sensors and Point of Care Diagnostics; Registries and Vital Events Tracking; Data Collection and Reporting; Electronic Health Records; Electronic Decision Support: Information, Protocols, Algorithms, Checklists; Provider-Provider Communication: User Groups, Consultation; Provider Work Planning and Scheduling; Provider Training and Education; Human Resource Management; Supply Chain Management; Financial Transactions and Incentives. It included studies which used SMS education, SMS reminders, voice calls, patient support by SMS, SMS campaings to improve treatment compliance, mobile-based light microscopy, tranmission of mobile phone pictures of dermatological conditions or cervices, mobile data collection tools, data transfering through SMS, SMS-based registration of birth rates, SMS exam results notification (SMS test reporting tool), verbal autopsies, mobile-based medical record system, SMS and voice support | Patients and healthcare practitioners | It varied among different studies. The information was not provided for all studies. | Health impacts were defined in terms of measurable changes in mortality, morbidity, disability adjusted life years (DALYs), and improved disease detection rates. Behaviour change was also included, as a valid health impact where changes in knowledge, self-efficacy, attitudes, or behaviours themselves had a reasonably direct association with improved health, such as improved antenatal care uptake, or reduced health personnel absenteeism |
| Aranda-Jan | 2014 | [12] | The 44 studies on mHealth projects in Africa were classified as: patient follow-up and medication adherence (n = 19), staff training, support and motivation (n = 2), staff evaluation, monitoring and guidelines compliance (n = 4), drug supply-chain and stock management (n = 2), patient education and awareness (n = 1), disease surveillance and intervention monitoring (n = 4), data collection/transfer and reporting (n = 10) and overview of mHealth projects (n = 2). Most of the mHealth projects focused on HIV, malaria, tuberculosis, diabetes and antenatal care. | Patients and professionals | Not reported | Patient folow-up and medication adherence (n=19); staff training, support and motivation (n=2); staff evaluation, monitoring and guidelines compliance (n=4); drug supply-chain and stock management (n=2); patients educationj and awareness (n=1); disease surveillance and intervention monitoring (n=4); data collection/transfer and reporting (n=10); overview of mHealth projects (n=2). Project sustainability: mid- and long term results and impacts. Project integration into the health system: relevance of the design, involvement of key stakeholders, compatibility to existing government policies and management information systems. Technology/existing infrastructure: cost, usage and acceptance, network coverage, electricity and other infrastructure. Project management process: related resources required for project implementation. Scale-up and replication: requirements for scaling-up projects at a regional or national level. Legal issues, regulations and standards: in-country regulations, laws or standards that influence mHealth projects. |

| First author | Year | Referenc e | Intervention | Target of intervention | Duration of follow up (months) | Outcomes |
|---|---|---|---|---|---|---|
| Beatty | 2013 | [13] | (1) Monitored exercise training (walking) 3 times weekly assisted by smartphone application. (2) 10 clinic supervised exercise sessions followed by 14 home exercise sessions with mobile application (3 sessions per week). (3) Lifestyle counseling, mobile intervention, devices for home monitoring. | Patients | 6 weeks - 12 months | Usability, participation, exercise capacity, health status, risk factors and events |
| Baron | 2012 | [14] | Patients were required to transmit blood glucose readings (and blood pressure readings, weight, exercise, diet, medication, free text, and/or their level of wellbeing in some studies) to an online server via a mobile device. Health care professional or automated feedback were given aiming to increase peer support, educate, or remind patients of appointments or self-care activities. Dietary management was an intervention in three studies of type 1 diabetes. | Patients | 3-12 months | Hb1Ac improvement |
| Guy | 2012 | [15] | Text messages to remind participants of their upcoming healthcare appointment. | Patient or patient parent/carer (in case of pediatric patients) | Not reported | Attendance rate (porportion of patients attending their appointment at the original schweduled time |
| Jongh *et al*. | 2012 | [16] | Interventions consisted exclusively in regular text messages (SMS or MMS) to facilitate self-management of long term illnesses: text messages with health information and medication (n=2), two-way communication between patients and an automated system with reminders for blood glucose monitoring with automated feedback/recommentations according to the glucose measures sent by the patien (n=1)t, and two-way communication between patients and healthcare providers with text messages to send daily asthma self-monitoring results and weekley feddback/advice (n=1). | Patients | 3 to 24 months | Primary: health outcomes (including physiological measures, biomarker values, self-reporting symptom resolution or quality of life), capacity to self-manage long-term conditions (life-style modification, understanding of th disease, impact on independence adn responsability, self-estemm and/or creation of supportive enviroment) Secondary: user (patient, carer or healthcare provider) evaluation of the intervention (satisfacion, readiness to use, timeliness, availabiliy and/or convenience) and perceptions of safety, healthcare utilization foloowing the intervention, costs (direct and indirect), potential harms and adverse effects. |
| Fanning | 2012 | [17] | Use of SMS (n=8), native mobile software (n=4) and/or personal digital assistant (PDA) (n=2) to provide SMS/mobile app/PDA self monitoring, tailored SMS/automated feedback, relay social support/reminders and motivational messaes/information about physical activities benefits/effective messages/implementation, and/or intention or goal reminders | Patients | Average 14.6 weeks (range 2-52 weeks) | Physical activity was measured through self-report and/or objective measures such as pedometer, accelerometers, the walking distance of the incremental shuttle walking test, and a mobile sensing platform. |

| First author | Year | Reference | Intervention | Target of intervention | Duration of follow up (months) | Outcomes |
|---|---|---|---|---|---|---|
| Gurol-Urganci | 2012 | [18] | Text messages to remind participants of their upcoming healthcare appointment. The texts were sent using web-based plataforms (n=3), automatically with a Global System Mobile (GSM) modem linked to an eletronic health records system; up to 24h before (n=3), 24-48h before (n=3), 72h before (n=1) the appointment or 4 messages per appointment (n=1). In 6 studies the delivery of the messages was verified by "message sent" prompts | All the patients that required appointment in the clinic/practice (n=7) or patients who failed 2 appointment within 1 year | Not reported | Primary: rate of attendance at healthcare appointment<br>Secondary: healthcare outcomes as a result of the intervention, costs (direct and indirect) of the intervention, user (patient, carer or healthcare provider), evaluation of the intervention, user perceptions of safety, potential harms or adverse effects |
| Vodopivec-Jamsek | 2012 | [19] | SMS/MMS: (i) to suport antenatal care of health pregnant women; (ii) to provide smoking cessation advice, support and distraction (plus Quit budies, TXT crave, TXT pulls and TXT quizzes); (iii) to provide medication reminders and reminding-reinforcing or reminding-correction message; (iv) to provide information on any of the 3 behaviours: pedometer usage, estimating beverage serving sizes and screening time (TV, video game, computers) with automatd feedback | Patients | 1 month to 26 weeks | Primary: all outcomes related to health status or health behaviour, such as adoption of healthier lifestyles (e.g. smoking cessation, increased physical activity, weight control, nutrition and stress management), or improved quality of life.<br>Secondary:health service utilization following the intervention, costs (direct na indirect) of the intervention, user (patient, carer or healthcare provider) evaluation of the intervention, user perceptions of safety, potential harms or adverse effects |
| Fjeldsoe | 2009 | [20] | SMS used for preventive health behaviour (n=4) or to support ongoing clinical care behaviour change (n=10) | Patients | 6 weeks to 6 months | HbA1C (n=6), peak flow (n=1), blood pressure (n=2), cout tables (n=1), short evaluation eating disorders (n=1), weight/waist circunference/BMI (n=1), positive change in physical activity (n=1), use/satisfaction/acceptance of the program (n=1), quit smoking orattempt to quit (n=2) |
| Whittaker | 2016 | [21] | Any type of mobile phone-based intervention for smoking cessation; imost of them were text messaging-based, although several paired text messaging with in-person visits or initial assessments. Two studies gave pre-paid mobile phones to low-income human immunodeficiency virus (HIV)-positive populations - one solely for phone counselling, the other also included text messaging. One study used text messages to link to video messages. The authors excluded trials where mobile phones were seen as an adjunct to face-to-face or Internet-based programmes, | Patients | 26 weeks for the primary outcome | Smoking abstinence at 26 weeks from the start of the intervention (analyzed overall and also separetly - continuous abstinence and 7-day point prevalence), 26-week biochemically verified cessation outcomes |
| Liang | 2011 | [22] | SMS and internet to initiate the intervention and provide support for self-monitoring blood glucose, continuous education, reinforcement of diet, exercise and medication adjustment (n=12); or short messages, SMS alone or combined with other intervention strategies, which included transmitting self-monitored blood glucose to mobile phone via Bluetooth wireless link (n=8); mobile phone-based intervention (e.g., encouraging self-monitoring of blood glucose, reinforcement of diet and exercise, and managing hypoglycaemia) (n=2); moblile phone interventions (i.e., self-monitoring of blood glucose support, and advice on drugs, food intake and activity level) combined with clinical visits that consisted of clinical advice and structured counselling from a diabetes specialist nurse. | Patients | 3 to 12 months | Hb1Ac improvement |

| First author | Year | Referenc e | Intervention | Target of intervention | Duration of follow up (months) | Outcomes |
|---|---|---|---|---|---|---|
| Bacigalupo | 2013 | [23] | Participants were educatedabout weight loss via diet and exercise, and carried with them in their waking hours a mobile device (i.e. a text pager, mobile phone or other) that was a motivator in behaviour change, such as by receiving a motivational message, recording what had been eaten and/or the amount of physical activity. | Patients | 9 to 52 weeks | Weight loss however measured and BMI |

## Supplementary file 2. Continued

| First author | Year | Reference | Main results | Lessons and barriers for implementation | Main limitations |
|---|---|---|---|---|---|
| Beratarrechea | 2014 | [1] | Positive impact on chronic disease outcomes, improvement on attendance rates, clinical outcomes, health-related quality of life. All studies about effect on healthcare costs found that mHealth is cost-effective. | The appropriateness of extrapolating the data found to the developing world context is unclear as the authors were only able to find articles from seven countries, most of them upper-middle-income countries and none of low-income countries. The integration of mobile interventions into the healthcare system in LMIC may be a feasible way to complement and improve strategies toward prevention and control of chronic diseases, but success in scaling up and sustainability depends on other factors besides mobile phone technology, such as the healthcare context, social values, and culture. In fact, public health and/or healthcare issues vary among LMIC. | The small number of RCTs using m-health to address chronic diseases in LMIC underscore the need of more rigorous implementation research on m-health in these countries; and the small study size of most of the studies included makes it difficult to interpret the applicability of study results to larger LMIC populations. |
| Peiris | 2014 | [2] | It was observed improvements in health care service delivery processes, behavior change (smoking cessation) and use of geographic information system to support improved health care. Effectiveness for clinical outcome (6), improvements in processes of care (3), cost (3), improvements in health-related quality of life (1), in clinical attendance rates (4). The most common diseases were diabetes or CVD and risk factors for CVD. Despite the promising findings demonstrated in this review, the authors concluded that the current evidence base is insufficient to guide decisions on policy and practice. | The current evidence base is insufficient to guide decisions on policy and practice. The authors recommend four priority areas to improve the mHealth research: (1) comparative effectiveness studies examining mHealth versus other “traditional” health care improvement strategies; (2) large, multinational studies powered on “hard” clinical endpoints such as mortality and hospitalizations that enable crosscountry comparisons; (3) process and economic evaluations of effective and failed interventions to determine contextual opportunities and constraints for scale-up; and (4) the need to examine policy-level barriers to largescale adoption of promising mHealth interventions. | Many studies lacked sufficient detail to characterize them in finer detail making difficult to appreciate differences between interventions within any particular mHealth domain. Owing to the paucity and heterogeneity of RCTs in the review, it was unable to conduct a quantitative meta-analysis of the outcomes. Although it was examined the leading contributors to NCD mortality, the authors did not examine other NCD areas such as musculoskeletal conditions which are a major contributor to disability. |
| Hamine | 2015 | [3] | SMS was the most commonly used mAdherence tool (40.2%), used to facilitate patient-provider communication, medication reminders and data collection and exchange on disease-specific measurements. The second tool used was specialized software or a smartphone app (23,4%), mainly among patients with Diabetes. A wireless or Bluetooth-compatible device was used in 17,8% of studies and a specific instrument connected to a phone, such as blood glucose meter in 13,1% of studies (also EKG, BP monitor, weighing machine). Usability, feasibility and acceptability or patient preferences for adherence interventions were assessed in 57,9% of studies and found to be generally high. From 27 RCTs that assessed impact on adherence behaviors, 15 (56%) observed significant improvements. From the 41 RCTs that measured effects on disease-specific clinical outcomes, significant improvements between groups were reported in 16 studies (39%). In general, mAdherence tools targeting low-income, elderly and minority groups were found to be usable with high satisfaction ratings. | Besides cost, language, and literacy barriers, availability and connectivity issues are also potential barriers to consider. Perhaps most critically, if adherence to chronic disease management is not encouraged and actively practiced, it is very unlikely that mAdherence will be successful. mHealth tools are communication platforms and delivery mechanisms, not solutions in and of themselves. mAdherence will only work where there is already a functioning adherence program in place. | It was not weighted the quality of evidence or study design against reported results. It was not include non-English literature, and some of the studies included as few as four participants. The diversity of study objectives, designs, and outcomes made clear comparisons difficult and the quality of evidence was variable. |
| Free (a) | 2013 | [4] | The trial demonstrated mixed evidence regarding the benefits of intervention. One trial with low risk of bias reported clinically important reductions in viral load from an intervention that used text messages with patients prescribed antiretroviral in Kenya. Trials demonstrated that support by text message more than doubles biochemically verified smoking cessation. There is suggestive evidence of benefit for reminders for vaccine appointment attendance and cardiopulmonary resuscitation training. There is suggestive evidence or short-term benefits for interventions for asthma control, physical activity, and psychological support interventions. The meta-analyses showed that to date, mobile technology-based interventions for diabetes control that have statistically significant effects are small and of borderline clinical importance. Simple medication reminders delivered by SMS message show no benefits. The effect estimates for diet and diet with physical activity interventions on weight were consistent with no or small benefits. | There is currently insufficient high quality evidence of beneficial effects on clinical outcomes to warrant implementation of interventions for other areas of health behaviour change or selfmanagement of diseases. The majority of the research to date has been conducted in high-income countries, so trials of interventions in low- and middle-income countries are required, particularly in view of the high coverage of mobile technologies in these settings. | One fourth of reviews did not provide sufficient data to calculate effect estimates and this could have resulted in bias in the systematic review findings. A wide range of factors could influence the effectiveness of mobile technology interventions including trial quality; participant factors such as demographics or disease status; the setting (low-/middle- or high-income country), intervention factors such as components, intensity, timing, type of mobile device; or the mobile technology function used. It was not possible to explore the factors influencing heterogeneity statistically, as there were few trials of similar interventions reporting the same outcomes, resulting in limited power for such analyses. The examination of funnel plots in exploring publication bias was limited. as few trials contributed to some pooled analyses. There was inadequate power to explore the impact of different behaviour change tools in specific behavioural domains on effect estimates. |

| First author | Year | Reference | Main results | Lessons and barriers for implementation | Main limitations |
|---|---|---|---|---|---|
| Agarwal | 2015 | [5] | With adequate training (from a few hours to 1 week), FHWs were able to use mobile phones to enhance various aspects of their work activities. Findings suggest that mobile based data collection improves promptness of data collection, reduces error rates and improves data completeness. Data collection is one of the primary mHealth functions being performed by FHWs. Two methodologically robust studies suggest that regular access to health information via SMS or mobile-based decision-support systems may improve the adherence of the FHW to treatment algorithms. Qualitative results: mobile-phone based tools was considered as an useful means to reinforce and improve the services provided and the users felt empowered and motivated and had more credibility in the community. The evidence on the effectiveness of the other approaches was largely descriptive and inconclusive. | Although enthusiastic about the potential of the use of mHealth tools by FHW, the literature on the effect of mobile phone-based alerts and reminders sent to FHW mobile phones, supervision and emergency referrals is still in its infancy and largely inconclusive. The biggest gap in knowledge about the use of mHealth strategies by FHWs at present is in the lack of evidence on how such strategies may improve health outcomes, health system efficiencies and cost-effectiveness of service delivery. | It was not include non-English literature and the quality of studies included was not assessed. The majority of studies were pilot activities with minimal information about the effectiveness, quality and efficiency of health systems functions and/or client health outcomes |
| Krishna | 2009 | [6] | Information and education interventions delivered through wireless mobile technology resulted in both clinical and process improvements in the majority of studies. Chronic diseases such as diabetes and asthma, requiring regular management, as well as smoking cessation requiring ongoing advice and support, benefited most from the cell phone interventions. Twenty of 25 studies (80%) reported significant diferences between control and intervention groups as a result of cell phone and text messaging intervention regardless of the frequency of message delivery. Fewer days to diagnosis and treatment were reported among those who were notified of test results via text messages. One study showed improvement in communication among persons with disabilities who were taught via text messaging. There was a significantly grater increase in compliance with medication taking among HIV-positive patients with memory impairment compared to those without impairment and with keeping hepatites A and B dose vaccination schedules among international travelers. There was also a significant improvement in insulin adherence (p<0,05) among persons with type 1 diabetes who received tailored text messages with goal-specific prompts. Diabetes education and advice via cell phone and text messaging resulted in significant reductions in HbA1c (p<0,05). Peak flow monitoring showed significantly greater improvements in asthma cough and night-time symptoms while lowering daily doses of medication. There was no significant difference between groups that did or did not receive alerts and reminders on how to control their blood pressure, participants in both groups had nearly equal percent of patientswith controlled blood pressure at follow-up. Significant decrease in anxiety score was described among those who received multimedia relaxation messages than those exposed to new age music or no intervention. A study of mobile phone personalized advice and motivational tips for physical activity observed a significant improvement (p<0,05) in percent body fat lost, however, body mass index, and systolic and diastolic blood pressure were unchanged. One study in the area of diabetes observed a significant improvement in quality of life (p<0,05) and satisfaction with life (p<0,05). Another diabetes study and a smoking cessation study observed significant improvement in self-efficacy (p<0,001 and p<0,01). | Healthcare providers should be willing to incorporate cell phone interventions which is a commom everyday technology. Therefore, studies are also needed on the cost-effectiveness and technical and financial feasibility of adoption in real clinical settings. | The small sample sizes, with two studies included in this review having less than 20 participants. The findings of these studies may not be generalizable to other populations. This review includes one study for which the authors only have access to a published English abstract and some information may have left out. Only two studies included evaluated cost. The reviewed studies did not express any concerns over the impediments to the use of cell phones such as lack of reimbursements to health professionals receiving the call, time commitment, or potential abuse of cell phone and SMS privilege. |
| Bloomfield | 2014 | [7] | (i) the study that analized the use of mobile phones by guardians of pediatric patients with Burkitt's lymphoma noted an increase (20% to 60%) in parents with mobile phone from 2007–2010 and 59% of parents were able to be contacted. However, there were no measured differences in clinical outcomes between those who could and could not be contacted. (ii) the mobile phone-based home glucose monitoring program study evidenced a decrease in HbA1C from 13.2% to 10.5% (P < 0.001) after 3–6 months. (iii) the system operated by non-physician health providers in a public sector cervical cancer prevention program concluded that referrals were feasible and bypassed historic barriers to care. (iv) the Nigerian teaching hospital that gave their oncologists' mobile phone numbers showed that 98% of patients contacted their oncologist, 73% of calls to discuss illness or treatment, 26% to arrange next appointment, 1% of calls to extend social greeting, and 100% of intervention patients and 19% of control patients adhered to clinic appointments. (v) the educational group sessions for diabetic women via SMS showed positive effects on sleep, positive action, and coping, more text messages between buddies than to the program (19883 vs.1321), 29% response rate to text questions, higher diastolic blood pressure (+7 mmHg) and less spiritual hope at 6 months, and high-texters had higher body mass index and more sedentary time. | Only a small number of mHealth strategies for NCDs have been studied in sub-Saharan Africa. The literature lacks controlled trials or other study designs with comparator arms and we found few mHealth deployments that incorporate monitoring or evaluation of effectiveness. | No limitation was described however non-English literature was not screaned, except when an abstract in english was available; as well as the quality of studies included was not assessed. |

| First author | Year | Reference | Main results | Lessons and barriers for implementation | Main limitations |
|---|---|---|---|---|---|
| Car | 2012 | [8] | (i) text message reminders improved the rate of attendance at healthcare appointments compared with no reminders (RR 1.10; 95% CI 1.03 to 1.17) and postal reminders (RR 1.10, 95% CI 1.02 to 1.19). However, text messages and phone reminders had similar effects on attendance (RR 0.99, 95% CI 0.95 to 1.03). (ii) two studies measured the cost per unit of effective intervention of text message versus telephone reminder and found that the relative cost of the text message reminder per attendance was 55% and 65% of the cost of phone call reminders. (iii) one study reported the pre-intervention acceptability of the intervention and foud that 98% of patients were willing to receive routine mobile phone text message reminders of their outpatient appointment. (iv) no adverse effects were reported in one study. None of the studies specifically reported events such as misreading or misinterpretation of data, transmission of innacurate data, loss of verbal and non-verbal communication cues, issues of privacy and disclosure, or failure or delay in the message delivery. (v) none of the included studies reported on health outcomes or user perception of safety. | As the review contains a relatively small number studies, it is difficult for authors to assess to what extent their findings have more general relevance. No consideration was given to issues of security and confidentiality by the studies included. Particularly in low-income countries where mobile phones are frequently shared between family members, these are important confidentiality issues that need to be taken into account when designing interventions using SMS. There is a lack of information about adverse effects and consumer satisfaction with the intervention. | By excluding studies with possible confounding from other communication and/or data transmission methods, the authors may have introduced selection bias towards less successful interventions, as more complex interventions may be more effective at improving attendance rates. The included studies were heterogeneous and the quality of the evidence therein is low to moderate, which makes the findings difficult to generalise. |
| Free (b) | 2013 | [9] | Seven trials of health care provider support reported 25 outcomes regarding appropriate disease management, of which 11 showed statistically significant benefits. One trial reported a statistically significant improvement in nurse/surgeon communication using mobile phones. Two trials that used mobile phones to transmit photos to off-site clinicians for diagnosis reported significant reductions in correct diagnoses compared to diagnosis by an on-site specialist. The pooled effect on appointment attendance using text message reminders versus no reminder was increased, with a relative risk (RR) of 1.06 (95% CI 1.05–1.07, $I^2$=6%). The pooled effects on the number of cancelled appointments was not significantly increased RR 1.08 (95% CI 0.89–1.30). There was no difference in attendance using SMS reminders versus other reminders (RR 0.98, 95% CI 0.94–1.02, respectively). | SMS messages are modestly effective as appointment reminders. Since their effects appear similar to other forms of reminder, health care providers should consider implementing SMS appointment reminders because the cost of missed appointments in health services is high, the cost of providing SMS appointment reminders is low, and SMS reminders are cheaper than other forms of reminder (e.g., a letter with stamp). Many of the interventions evaluated to date are single component interventions of low intensity and effects of higher intensity multi-component mobile technology interventions should be evaluated. Interventions combining elements delivered by mobile technology with other treatments such as clinics based counselling combined with text messages should be systematically reviewed. | The authors reported that was not appropriate to pool the results as the interventions targeted different diseases and outcomes. Further, there are likely to be important differences in the intervention content of interventions (such as the behaviour change techniques used), even in those using the same mobile technology functions (such as application software). It was not possible to explore how different intervention components influenced outcomes as the intervention components were not described consistently or in detail in the authors' papers and also it was not possible to explore how the intervention components targeting the disease and outcomes influenced the results. |
| Valles-Ortiz | 2015 | [10] | A significant reduction in HbA1c levels was observed in 6 of the 8 studies, although the period of duration of interventions was not the same for all studies. Two of the studies provided support through phone calls; another study utilized a video of care methods and the remaining 5 mention sending messages with educational and motivational content. From all this information it can be inferred that there was no uniformity among interventions to demonstrate that sending SMS exclusively is an effective strategy for maintaining type 2 diabetes patients' glycemic control. | It is necessary to conduct more studies on this option before generalizing its utilization by most people in type 2 diabetes | Inclusion and exclusion criteria were not completely clear, and the authors did not provide the results of quality assessment or conflicts of interest. Sample size was small for the majority of studies. Meta-analysis was not performed. |
| Hall | 2014 | [11] | Client education and behaviour change (20 studies): (1) Conflicting evidence about improving tuberculosis medication adherence; (2) Evidence of improvement of HIV anti-retroviral adherence, except form a Chinese study that used voice call, but did not improve uptake of HIV treatment; (3) Mixed and scant evidence with interventions that target non-communicable diseasesFirst, client-based interventions for education and behaviour change are a rapidly increasing area of interest, now that substantial proportions of people in many LMIC populations have access to mobile communications technology. SMS reminder systems for appointments have improved appointment adherence, which is likely to improve service quality and efficiency of health systems. The second major grouping is largely technical; applications involving imaging, data collection, registration procedures, and patient records may well benefit from mHealth components, but also need wider functionality in health systems to be beneficial. The third major area is in mHealth tools that directly support health workers. These range across domains that provide information and decision support to professionals, which may be beneficial in terms of technical and managerial issues, enable more effective communication with clients, and directly enhance logistic issues such as supply chains. No results about human resource management and finacial transactions and incentives. | Limited access to mobile phones by women in some countries, such as Uganda | Although the authors performed a systematic search, they did not perform a systematic review. They did not report how study selection and data extraction was performed and did not perform quality assessment of included studies. |

| First author | Year | Referenc e | Main results | Lessons and barriers for implementation | Main limitations |
|---|---|---|---|---|---|
| Aranda-Jan | 2014 | [12] | The majority of the studies reported successes and positive outcomes of mHealth in Africa. There were positive results in (1) Implementations to improve life-style medication adherence and treatment follow-up. The feasibility ad potention of mHealth implementations is unanimosly agreed upon across the studies. Oucomes are not always consistent bewween pilot projetcs and RCTs/mixed-methods studies; (2) Staff training, support and motivation, mostly for community health workers (Uaganda, South Africa and Botswana) ; (3) Staff evaluation, monitoring and compliance to guidelines, with evidence of improvement of quality of care in Kenya and Uganda; (4) Improving drug supply chain in Kenya and Tanzania, reducing out-of-stock and supporting drug stock management (pilot studies); (5) Disease surveillance and monitoring, mostly malaria, in Zambia and Uganda. In Rwanda, the researchers found mobile data collection logistically complex and time consuming; (6) Data collection - use of SMS proved to be feasible for delivery of information in real time, to imporve information qaulity, reduce data losses ad reporting errors and reduce data uploading difficulties; (7) health education and awareness - it is shown to be feasible, but there is lack of evience of impact. | MHealth integration into the healthcare system is critical to achieve the maximum benefits.The participation of the government is a fundamental aspect for success. Projects have proved to be successful when they have been adapted to local context and language, and when the project has been implemented by public-private partnership. Planning costs and logistics are essencial. Ease-of-use, familiarity with and access to the technology are important factors for implementation success. User's literacy and high worload for health workers are barriers. High phone sharing, lack of money to top-up a phone and male control over a household phone ownership may also limit the results of a project. Major threats to mHealth porjects include cultural perception, lanuage, limited resources n rural settings, weak health systems and externam financial schemes | Quality of the systematic review ( included only studies published in English, peer-reviewed,;risk of bias of included studies was not accessed and detailed information in each study was not provided). The authors state that one of the major weaknesses of studies in mHealth projects is that claimed benefits are unclear and long-term results remain uncertain. |
| Beatty | 2013 | [13] | (i) Usability: 80% of sessions no technical problems. Ease of use rated 4.8/5 (95% CI 4.6 to 5.0). Participation: Completed 80% of scheduled exercise sessions. Exercise Capacity: 6 minute walk test improved from 524 to 637 m (P=0.009). Health Status: SF-36 (Short Form Health Survey) Physical Health increased from 50.0 to 78.4 (P=0.03). Mental Health unchanged. (ii) Exercise Capacity: 17.6 +- 16.1% improvement mobile vs 11.5 +- 35.9% control (P>0.05). Risk Factors: blood pressure not significantly changed in either group. (iii) Usability: mHealth group completed 89% of entries. 5/102 dropped out due to difficulty with mHealth intervention. Physical Activity: 75% met goals in mHealth group vs 73% control. Risk Factors: mHealth group more likely to improve at least 1 risk factor (RR 1.4, 95% CI 1.1 to 1.7). mHealth group more likely to achieve goals for blood pressure (62.1% vs 42.9%), HbA1c (86.4% vs 54.2%), and body mass index (0.37 kg/m$^2$ decrease vs 0.38 increase). No significant differences in smoking cessation, cholesterol, medication adherence. Events: 5 deaths in control group, 0 in mHealth group. | Initialevidence supports the feasibility and usability of using mobile technology for cardiac rehabilitation for patients with ischemic heart disease. However, further studies are necessary to access whether using mobile technology can improve access, increase participation and improve outcomes in patients with ischemic eart disease. the authors proposed a framework for the development and evaluation of mobille applications for cardiac rehabilitation for patients with ischemic heart disease. | Quality of the systematic review (only one databased searched, search terms could be improved, included only studies published in English, only 1 author decided about study inclusion, risk of bias of included studies was not accessed. Onyl 3 studies were included, all of them in high-income countries |
| Baron | 2012 | [14] | Type 1 diabetes: for studies evaluating a diet-focused intervention results were mixed, and non-dietary interventions were inconclusive with 2 of the 4 studies supporting the effectiveness of mhealth. Type 2 diabetes: mhealth was found to be effective for people with poorly controlled diabetes while also being more effective than standard care in helping people with well-controlled diabetes maintain glycemic control. Ten of the 13 studies in type 2 diabetes and 4 of 7 studies on type 1 diabetes found mhealth to lead to benefits. Studies without health care professionals feedback led to improved HbA1c, suggesting professionals feedback might not be necessary for intervention success. The recording and tracking of data could be the key factor for increasing patients' awareness, understanding, and motivation to self-manage. Knowledge that the data are accessible to health care professionals may also be an incentive to adhere to a regimen. The graphical and automated text feedback might also be an effective incentive to engage patients. None pf the studies fuond mhealth to be harmful. | The recording and tracking of data could be the key factor for increasing patient's awareness, understanding and motivation to self-manage. | The methodological quality of included studies was poor, with many involving small sample sizes, no power calculations and poor study designs. Many studies assessed intervention efficacy and not effectiveness, as they excluded patients who failed to engage with the devices from the analysis (per protocol analysis). The costs incurred in the delivery and running of these telemonitoring interventions as not discussed in this review. |
| Guy | 2012 | [15] | There was significant heterogeneity in the estimated effect measure of the relationship between use of SMS and clinic attendance due to the observational studies. The summary effect from the RCTs was 1.48 (95% CI 1.23-1.72) with no significant subgroup differences (clinic type, message timing and target age group). | Short message service reminders subsequently increase the likelihood of attending clinic appointments. Further research is necessary to assess whether it improves clinical outcomes. | It is possible that some studies were not identified in the search, particularly those with a negative outcome (although there was no evidence of publication bias by the funnl plot). The unit of analysis was the consultation and not the patient: patients who attende an appointment are more likely to attend a subsequent appointment, while those who fail to atted the first appointment may not have another apportunity. Methodological limitations of the systematic review. |

| First author | Year | Referenc e | Main results | Lessons and barriers for implementation | Main limitations |
|---|---|---|---|---|---|
| Jongh *et al.* | 2012 | [16] | Diabetes: moderate quality evidence (2 studies) showing no statistical difference from text messaging interventions compared to usual care or email for glycemic control (HbA1C), the frequency of diabetic complications or body weight. Hypertension: moderate quality evidence (1 study) that the mean blood pressure, the proportion of patients who achieved blood pressure control and mean body weight were not significantly different in intervention and control groups. Asthma: moderate quality evidence (1 study) of greater improvements on peak expiratory flow variability (mean difference -11.12, 95% CI -19.56 to -2.68) and symptoms(pooled score of cought, night symptoms, sleep quality and maximum tolerated activity) (mean difference -0.36, 95% CI -0.56 to -0.17) compared to the control group, with no difference in impact on forced vital capacity or forced expiratory flow in 1 second.<br>Secondary outcomes: (1) Moderate quality evidence from 1 study that diabetes patients receiving the textmessaging intervention demonstrated improved scores onmeasures of self-management capacity, but did not show improved knowledge of diabetes; (2) Moderate quality evidence from 3 studies of the effects on treatment compliance: conflicting evidence about patients' rates of medication compliance and no statistically significant effect on rates of compliance with peak expiratory flow measurement in asthma patients, text message prompts for diabetic patients initially resulted in a higher number of blood glucose results sent back (46.0) than email prompts did (23.5); (3) Very low quality evidence (2 studies) of perceived improvement in diabetes self-management, desire to continue receiving messages, and preference of mobile phone messaging to email as a method to access a computerised reminder system, (4) Very low quality evidence from 2 studies that diabetes patients receiving text messaging support made a comparable number of clinic visits and calls to an emergency hotline as patients without the support. For asthma patients the total number of office visits was higher in the text messaging group, whereas the number of hospital admissions was higher for the control group. | There are significant information gaps regarding long-term effects, acceptability, costs and rcountries.isks of such interventions, as well as the impact in low-incom | The authors included only studies in which the intervention is delivered exclusively through text messaging. This strategy restricted the body of evidence the authors were able to build on: only 4 studies with sample size no larger than 67 patients. So, it is very difficult<br>to assess the external validity. The short follow-up time (up tof 12 months) means that no conclusions can be drawn about the long-term effects. All of the included studies were set in high-income countries where mobile phone ownership is widespread and data transmission reliable. None of the studies evaluated potential complications, such as loss or misinterpretation of data.No consideration was given to issues of security and confidentiality. Particularly in low-income countries where mobile phones are frequently shared between family members, these are important issues that need to be taken into account. Eight trials were ongoing at time of publication (2012) |
| Fanning | 2012 | [17] | There was significant moderate to large effect for pedometer steps (g = 1.05, 95% CI = 0.75 to 1.35, P < .01). Interventions delivered via mobile phone yielded a significant moderate effect (g =.52, 95% CI = 0.11 to .94, P= .01). The effects were non-significant for both MVPA duration (g = 0.20, 95% CI = -0.19 to 0.60, P= .31) as well as for PDA delivered (g = .68, 95% CI = -0.88 to 2.25, P = .39), with lacking significance in the latter likely due in large part to the small number of studies and considerable heterogeneity. | Few studies report on key internal (eg, delivery as intended) or external (eg, descriptions of participants, settings, and delivery staff) factors. As a result, the degree to which these findings are robust and generalizable cannot be determined.(usar estudo do Blackman como referência) | Small number of published studies necessitated broad inclusionary criteria, thereby including studies that varied greatly in population characteristics, study design, and use of mobile components |
| Gurol-Urganci | 2012 | [18] | Moderate quality evidence from 7 studies (5841 participants) that text message reminders improved the rate fo attendance compared to no reminders (RR 1.14 95% CI 1.03-1.26), and moderate quality evidence from 3 studies that text message reminders had a similar impact to phone call reminders (RR 0.99 95% CI 0.95-1.02). Low quality evience form 1 study (291 participants) suggests that text message reminders improved the rate fo attendance compared to postal reminders (RR 1.10 95% CI 1.02-1.19). Two studies reported that text messages reminders are more cost-effective than phone call reminders. | The current evidence still remains insufficient to conclusively inform policy decisions. Lack of information about health effects, adverse effects and harms, user evaluation of the intervention, user perceptions of its safety and cost-effectiveness | Studies covered low-income, middle-income and high-income countries. However, as the review contains a relatively limited number of studies, the authors consider difficult to assess to what extend these findings can be generalized |

| First author | Year | Reference | Main results | Lessons and barriers for implementation | Main limitations |
|---|---|---|---|---|---|
| Vodopivec-Jamsek | 2012 | [19] | Primary outcomes: Moderate quality evidence (1 study) that women who received prenatal support via text messages had significantly higher satisfaction than those who did not receive the messages, in the antenatal (mean difference 1.25, 95% CI 0.78 to 1.72) and perinatal period (mean difference 1.19, 95% CI 0.37 to 2.01); higher confidence level (mean difference 1.12, 95% CI 0.51 to 1.73) and lower anxiety level (mean difference -2.15, 95% CI -3.42 to -0.88) than in the control group in the antenatal period. Low quality evidence that text messaging intervention did not affect pregnancy outcomes (gestational age at birth, infant birth weight, preterm delivery and route of delivery). Moderate quality evidence (1 study) that mobile phone message reminders to take vitamin C for preventive reasons resulted in higher adherence (risk ratio (RR) 1.41, 95% CI 1.14 to 1.74). High quality evidence (1 study) that participants receiving mobile phone messaging support had a significantly higher likelihood of quitting smoking than those in a control group at 6 weeks (RR 2.20, 95% CI 1.79 to 2.70) and at 12 weeks follow-up (RR 1.55, 95% CI 1.30 to 1.84). At 26 weeks, there was only a significant difference between groups if, for participants with missing data, the last known value was carried forward. Very low quality evidence (1 study) that mobile phone messaging interventions for self-monitoring of healthy behaviours related to childhood weight control did not have a statistically significant effect on physical activity, consumption of sugar-sweetened beverages or screen time.<br>Secondary outcomes: Very low quality evidence (1 study) that user evaluation of the intervention was similar between groups. Moderate quality evidence (1 study) of no difference in adverse effects of the intervention, measured as rates of pain in the thumb or finger joints, and car crash rates. None of the studies reported health service utilisation or costs of the intervention | The acceptability of text messaging in preventive healthcare is an area that requires further attentionm, as well as evaluation of costs. | The authors included only studies in which the intervention is exclusively delivered through text messaging, and communication is between provider and participant only. This strategy restricted the body of evidence that they were able to examine.The small number of studies and the substantial heterogeneity in the selected studies made it difficult to assess to what extent the review's findings have more general relevance. No data has been collected beyond a study period of 26 weeks, so it is difficult to predict the long-term effects. As the review contains only one study for each individual field of primary prevention, it is very difficult to assess to what extent our findings have more general relevance. None of the studies evaluated potential loss or misinterpretation of the data, or considered issues of costs, security and confidentiality. |
| Fjeldsoe | 2009 | [20] | Of the 14 studies, 13 reported positive behaviour changes, although some studies were too statistically underpowered to show significant results. | It is important ot improve the quality and rigor of future resarch in this area. Further research is necessary on the effects of specific SMS characteristics. | The broad range of study designs and the varying use of specific SMS characteristics in interventions imit the conclusions that can be drawn. In somes studies, t was difficut to determine the relative impact of SMS because it was evaluated as na adjunct rather than a comprehensive strategy. |
| Whittaker | 2016 | [21] | All 12 studies pooled using their most rigorous 26-week measures of abstinence provided an RR of 1.67 (95% CI 1.46 to 1.90; I2 = 59%). Six studies verified quitting biochemically at six months (RR 1.83; 95% CI 1.54 to 2.19). | In high-income countries with existing tobacco control policies, media and education, text message-based smoking cessation interventions, either alone or in combination with face-to-face assessments or online programmes, appear to be a helpful option to offer to quitters. It is not yet clear whether this translates to low- or middle-income countries, and younger people. | All of the studies included were conducted in high-income countries with mature tobacco control policies; although two studies specifically recruited from low-income populations |
| Liang | 2011 | [22] | There was significant reduction of HbA1c values by a mean of 0.5% [6 mmol⁄mol; 95% confidence interval, 0.3–0.7% (4–8 mmol⁄mol)] in mobile phone interventions for diabetes self-management groups over a median of 6 months follow-up duration. In subgroup analysis, 11 studies among Type 2 diabetes patients reported significantly greater reduction in HbA1c than studies among Type 1 diabetes patients [0.8% (9 mmol⁄mol) versus 0.3% (3 mmol⁄mol); p = 0.02]. There was no significant effect of mobile phone intervention by other participant characteristics or intervention strategies. | Mobile phone interventions may not be suitable for all patients with diabetes. There may have drop outs due to opearting technical problems or intrusion into patients' life. Some withdraws reported in the studies included were the patient cannot use the special cell phone on a regular day-to-day basis or do not have computer and Internet access, which is a limitation of the widespread use of mobile phone interventions, especially by low-income patients. | Important confounding and selection bias may remain in the original studies. For instance, patients enrolled in a mobile phone intervention study may be more likely to show reductions in HbA1c values because of motivation. The authors found that smaller studies reported greater reduction in HbA1c values than did larger studies, which possibly reflected publication bias, with smaller trials more likely to report and publish their results if they found strong effects. There is no gold standard of calculating the missing standard deviation, and therefore random errors may exist in the imputations. |

| First author | Year | Referenc e | Main results | Lessons and barriers for implementation | Main limitations |
|---|---|---|---|---|---|
| Bacigalupo | 2013 | [23] | Five of seven trials reported greater weight loss in the intervention groups, of which two trials meet the recommended clinical standard of 5-10% weight loss, but only one had low risk of bias. | Practical boundaries concerning portability are constantly changing. Future research is needed and must involve well-designed high-quality RCTs that report results that facilitate meta-analyses, evaluation of the long-term benefit and cost-effectiveness of interventions employing mobile technology | The search of the terms was limited to title/abstract fields, then some terms could have been missed in the search strategy if they were not mentioned in these fields. |

BP, blood pressure; CVD, cardiovascular disease; FHWs, Frontline Health Workers; EKG, electrocardiogram; HRQoL, health related quality of life; LMIC, low and middle income countries, N, no; PDA, personal digital assistants; RCT, randomized controlled trial; SMS, short message service

## REFERENCES


1. Beratarrechea A, Lee AG, Willner JM, Jahangir E, Ciapponi A, Rubinstein A. The impact of mobile health interventions on chronic disease outcomes in developing countries: a systematic review. Telemedicine journal and e-health : the official journal of the American Telemedicine Association. 2014 Jan;20(1):75-82. PMID: 24205809. doi: 10.1089/tmj.2012.0328.

2. Peiris D, Praveen D, Johnson C, Mogulluru K. Use of mHealth systems and tools for non-communicable diseases in low- and middle-income countries: a systematic review. Journal of cardiovascular translational research. 2014 Nov;7(8):677-91. PMID: 25209729. doi: 10.1007/s12265-014-9581-5.

3. Hamine S, Gerth-Guyette E, Faulx D, Green BB, Ginsburg AS. Impact of mHealth chronic disease management on treatment adherence and patient outcomes: a systematic review. Journal of medical Internet research. 2015;17(2):e52. PMID: 25803266. doi: 10.2196/jmir.3951.

4. Free C, Phillips G, Galli L, Watson L, Felix L, Edwards P, et al. The effectiveness of mobile-health technology-based health behaviour change or disease management interventions for health care consumers: a systematic review. PLoS medicine. 2013;10(1):e1001362. PMID: 23349621. doi: 10.1371/journal.pmed.1001362.

5. Agarwal S, Perry HB, Long LA, Labrique AB. Evidence on feasibility and effective use of mHealth strategies by frontline health workers in developing countries: systematic review. Tropical medicine & international health : TM & IH. 2015 Aug;20(8):1003-14. PMID: 25881735. doi: 10.1111/tmi.12525.

6. Krishna S, Boren SA, Balas EA. Healthcare via cell phones: a systematic review. Telemedicine journal and e-health : the official journal of the American Telemedicine Association. 2009 Apr;15(3):231-40. PMID: 19382860. doi: 10.1089/tmj.2008.0099.

7. Bloomfield GS, Vedanthan R, Vasudevan L, Kithei A, Were M, Velazquez EJ. Mobile health for non-communicable diseases in Sub-Saharan Africa: a systematic review of the literature and strategic framework for research. Globalization and health. 2014;10:49. PMID: 24927745. doi: 10.1186/1744-8603-10-49.

8. Car J, Gurol-Urganci I, de Jongh T, Vodopivec-Jamsek V, Atun R. Mobile phone messaging reminders for attendance at healthcare appointments. The Cochrane database of systematic reviews. 2012 (7):CD007458. PMID: 22786507. doi: 10.1002/14651858.CD007458.pub2.

9. Free C, Phillips G, Watson L, Galli L, Felix L, Edwards P, et al. The effectiveness of mobile-health technologies to improve health care service delivery processes: a systematic review and meta-analysis. PLoS medicine. 2013;10(1):e1001363. PMID: 23458994. doi: 10.1371/journal.pmed.1001363.

10. Valles Ortiz PM, Miranda Felix P, García Sosa ES. Mensajes de texto para el control glucémico en adultos con diabetes tipo 2: revisión sistemática. Enfermería Global. 2015;14:435-44.

11. Hall CS, Fottrell E, Wilkinson S, Byass P. Assessing the impact of mHealth interventions in low- and middle-income countries--what has been shown to work? Global health action. 2014;7:25606. PMID: 25361730. doi: 10.3402/gha.v7.25606.

12. Aranda-Jan CB, Mohutsiwa-Dibe N, Loukanova S. Systematic review on what works, what does not work and why of implementation of mobile health (mHealth) projects in Africa. BMC public health. 2014;14:188. PMID: 24555733. doi: 10.1186/1471-2458-14-188.

13. Beatty AL, Fukuoka Y, Whooley MA. Using mobile technology for cardiac rehabilitation: a review and framework for development and evaluation. Journal of the American Heart Association. 2013;2(6):e000568. PMID: 24185949. doi: 10.1161/JAHA.113.000568.

14. Baron J, McBain H, Newman S. The impact of mobile monitoring technologies on glycosylated hemoglobin in diabetes: a systematic review. Journal of diabetes science and technology. 2012 Sep;6(5):1185-96. PMID: 23063046.

15. Guy R, Hocking J, Wand H, Stott S, Ali H, Kaldor J. How effective are short message service reminders at increasing clinic attendance? A meta-analysis and systematic review. Health services research. 2012 Apr;47(2):614-32. PMID: 22091980. doi: 10.1111/j.1475-6773.2011.01342.x.

16. de Jongh T, Gurol-Urganci I, Vodopivec-Jamsek V, Car J, Atun R. Mobile phone messaging for facilitating self-management of long-term illnesses. The Cochrane database of systematic reviews. 2012;12:CD007459. PMID: 23235644. doi: 10.1002/14651858.CD007459.pub2.

17. Fanning J, Mullen SP, McAuley E. Increasing physical activity with mobile devices: a meta-analysis. Journal of medical Internet research. 2012;14(6):e161. PMID: 23171838. doi: 10.2196/jmir.2171.

18. Gurol-Urganci I, de Jongh T, Vodopivec-Jamsek V, Atun R, Car J. Mobile phone messaging reminders for attendance at healthcare appointments. The Cochrane database of systematic reviews. 2013 (12):CD007458. PMID: 24310741. doi: 10.1002/14651858.CD007458.pub3.

19. Vodopivec-Jamsek V, de Jongh T, Gurol-Urganci I, Atun R, Car J. Mobile phone messaging for preventive health care. The Cochrane database of systematic reviews. 2012;12:CD007457. PMID: 23235643. doi: 10.1002/14651858.CD007457.pub2.

20. Fjeldsoe BS, Marshall AL, Miller YD. Behavior change interventions delivered by mobile telephone short-message service. Am J Prev Med. 2009 Feb;36(2):165-73. PMID: 19135907. doi: 10.1016/j.amepre.2008.09.040.

21. Whittaker R, McRobbie H, Bullen C, Rodgers A, Gu Y. Mobile phone-based interventions for smoking cessation. The Cochrane database of systematic reviews. 2016;4:CD006611. PMID: 27060875. doi: 10.1002/14651858.CD006611.pub4.

22. Liang X, Wang Q, Yang X, Cao J, Chen J, Mo X, et al. Effect of mobile phone intervention for diabetes on glycaemic control: a meta-analysis. Diabetic medicine : a

journal of the British Diabetic Association. 2011 Apr;28(4):455-63. PMID: 21392066. doi: 10.1111/j.1464-5491.2010.03180.x.

23. Bacigalupo R, Cudd P, Littlewood C, Bissell P, Hawley MS, Buckley Woods H. Interventions employing mobile technology for overweight and obesity: an early systematic review of randomized controlled trials. Obesity reviews : an official journal of the International Association for the Study of Obesity. 2013 Apr;14(4):279-91. PMID: 23167478. doi: 10.1111/obr.12006.
