# Supplementary material for: The Impact of mHealth Interventions: Systematic Review of Systematic Reviews
Source: JMIR Mhealth Uhealth. 2018 Jan 17;6(1):e23. doi: 10.2196/mhealth.8873 (PMC5792697; doi:10.2196/mhealth.8873)
Supplement: Multimedia Appendix 3 [file mhealth_v6i1e23_app3.pdf]

Supplementary file 3. Quality assessment ratings of systematic reviews included in mHealth review

Author, year	Reference	AMSTAR score											AMSTAR score Summary				Funding or support for the systematic review
		ed?Was an 'a priori' design	?Was there duplicate study selection and data	ned?Was a comprehensive literature search	erion? Was the status of publication (i.e. grey literature) used as an	ed? Was a list of studies (included and excluded)	?Were the characteristics of the included studies	?Was the scientific quality of the included studies assessed and	?Was the scientific quality of the included studies used appropriately in formulating	?Were the methods used to combine the findings of	Was the likelihood of publication bias assessed?	?Was the conflict of interest	No	Yes	Can't answer	Not applicable	
Agarwal *et al*., 2015	[1]	n	ca	y	y	n	n	n	na	na	n	n	6	2	1	2	Yes
Aranda-Jan *et al*., 2014	[2]	n	y	n	n	n	n	n	n	na	na	n	8	1	0	2	Unclear/not stated
Bacigalupo *et al*., 2013	[3]	n	y	y	y	y	y	y	y	na	na	n	2	7	0	2	Yes
Baron *et al*., 2012	[4]	n	n	n	n	n	y	y	y	na	n	n	7	3	0	1	Unclear/not stated
Beatty *et al*., 2013	[5]	n	n	n	n	n	n	n	n	na	na	n	9	0	0	2	No
Beratarrechea *et al*., 2014	[6]	n	y	y	y	n	n	y	n	na	n	n	6	4	0	1	Yes
Bloomfield *et al*., 2014	[7]	y	y	y	y	y	y	n	na	na	n	n	3	6	0	2	No
Car *et al*., 2012	[8]	y	y	y	y	y	y	y	y	y	y	n	1	10	0	0	Yes
de Jongh *et al*., 2012	[9]	y	y	y	y	y	y	y	y	y	na	n	1	9	0	1	Yes
Fanning *et al*., 2012	[10]	n	y	n	n	n	y	y	y	y	n	n	6	5	0	0	Unclear/not stated
Fjeldsoe *et al*., 2009	[11]	n	y	n	n	n	n	n	na	na	na	n	7	1	0	3	No
Free *et al*., 2013	[12]	y	y	y	y	y	y	y	y	y	y	n	1	10	0	0	No
Free *et al*., 2013	[13]	y	y	y	y	y	y	y	y	y	y	n	1	10	0	0	No
Gurol-urganci *et al*., 2012	[14]	y	y	y	y	y	y	y	y	y	n	y	1	10	0	0	No
Guy *et al*., 2012	[15]	n	ca	y	y	y	n	n	na	y	y	n	4	5	1	1	No
Hall *et al*., 2014	[16]	n	n	n	y	n	n	n	na	na	n	n	8	1	0	2	No
Hamine *et al*., 2015	[17]	n	ca	n	y	n	y	n	na	na	n	n	6	2	1	2	Unclear/not stated
Krishna *et al*., 2009	[18]	n	ca	n	n	n	n	n	na	na	n	n	8	0	1	2	No
Liang *et al*., 2011	[19]	n	y	y	y	n	n	y	y	y	n	n	5	6	0	0	Yes

Author, year	Reference	AMSTAR score											AMSTAR score Summary				Funding or support for the systematic review
		ed?Was an 'a priori' design	?Was there duplicate study selection and data	ned?Was a comprehensive literature search	erion? Was the status of publication (i.e. grey literature) used as an	ed? Was a list of studies (included and excluded)	?Were the characteristics of the included studies	?Was the scientific quality of the included studies assessed and	?Was the scientific quality of the included studies used appropriately in formulating	?Were the methods used to combine the findings of	Was the likelihood of publication bias assessed?	?Was the conflict of interest	No	Yes	Can't answer	Not applicable	
Peiris *et al*., 2014	[20]	n	y	y	y	y	y	y	n	na	n	n	4	6	0	1	No
Valles Ortiz *et al*., 2015	[21]	y	ca	y	y	n	y	n	na	na	n	n	4	4	1	2	Unclear/not stated
Vodopivec-Jamsek *et al*., 2012	[22]	y	y	y	y	y	y	y	y	na	na	n	1	8	0	2	Yes
Whittaker *et al*., 2016	[23]	y	y	y	y	y	y	y	y	y	n	n	2	9	0	0	Unclear/not stated

n, no; y, yes; ca, can not answer; na, not applicable.

REFERENCES

1. Agarwal S, Perry HB, Long LA, Labrique AB. Evidence on feasibility and effective use of mHealth strategies by frontline health workers in developing countries: systematic review. Tropical medicine & international health : TM & IH. 2015 Aug;20(8):1003-14. PMID: 25881735. doi: 10.1111/tmi.12525.

2. Aranda-Jan CB, Mohutsiwa-Dibe N, Loukanova S. Systematic review on what works, what does not work and why of implementation of mobile health (mHealth) projects in Africa. BMC public health. 2014;14:188. PMID: 24555733. doi: 10.1186/1471-2458-14-188.

3. Bacigalupo R, Cudd P, Littlewood C, Bissell P, Hawley MS, Buckley Woods H. Interventions employing mobile technology for overweight and obesity: an early systematic review of randomized controlled trials. Obesity reviews : an official journal of the International Association for the Study of Obesity. 2013 Apr;14(4):279-91. PMID: 23167478. doi: 10.1111/obr.12006.

4. Baron J, McBain H, Newman S. The impact of mobile monitoring technologies on glycosylated hemoglobin in diabetes: a systematic review. Journal of diabetes science and technology. 2012 Sep;6(5):1185-96. PMID: 23063046.

5. Beatty AL, Fukuoka Y, Whooley MA. Using mobile technology for cardiac rehabilitation: a review and framework for development and evaluation. Journal of the American Heart Association. 2013;2(6):e000568. PMID: 24185949. doi: 10.1161/JAHA.113.000568.

6. Beratarrechea A, Lee AG, Willner JM, Jahangir E, Ciapponi A, Rubinstein A. The impact of mobile health interventions on chronic disease outcomes in developing countries: a systematic review. Telemedicine journal and e-health : the official journal of the American Telemedicine Association. 2014 Jan;20(1):75-82. PMID: 24205809. doi: 10.1089/tmj.2012.0328.

7. Bloomfield GS, Vedanthan R, Vasudevan L, Kithei A, Were M, Velazquez EJ. Mobile health for non-communicable diseases in Sub-Saharan Africa: a systematic review of the literature and strategic framework for research. Globalization and health. 2014;10:49. PMID: 24927745. doi: 10.1186/1744-8603-10-49.

8. Car J, Gurol-Urganci I, de Jongh T, Vodopivec-Jamsek V, Atun R. Mobile phone messaging reminders for attendance at healthcare appointments. The Cochrane database of systematic reviews. 2012 (7):CD007458. PMID: 22786507. doi: 10.1002/14651858.CD007458.pub2.

9. de Jongh T, Gurol-Urganci I, Vodopivec-Jamsek V, Car J, Atun R. Mobile phone messaging for facilitating self-management of long-term illnesses. The Cochrane database of systematic reviews. 2012;12:CD007459. PMID: 23235644. doi: 10.1002/14651858.CD007459.pub2.

10. Fanning J, Mullen SP, McAuley E. Increasing physical activity with mobile devices: a meta-analysis. Journal of medical Internet research. 2012;14(6):e161. PMID: 23171838. doi: 10.2196/jmir.2171.

11. Fjeldsoe BS, Marshall AL, Miller YD. Behavior change interventions delivered by mobile telephone short-message service. Am J Prev Med. 2009 Feb;36(2):165-73. PMID: 19135907. doi: 10.1016/j.amepre.2008.09.040.

12. Free C, Phillips G, Galli L, Watson L, Felix L, Edwards P, et al. The effectiveness of mobile-health technology-based health behaviour change or disease management interventions for health care consumers: a systematic review. PLoS medicine. 2013;10(1):e1001362. PMID: 23349621. doi: 10.1371/journal.pmed.1001362.

13. Free C, Phillips G, Watson L, Galli L, Felix L, Edwards P, et al. The effectiveness of mobile-health technologies to improve health care service delivery processes: a systematic review and meta-analysis. PLoS medicine. 2013;10(1):e1001363. PMID: 23458994. doi: 10.1371/journal.pmed.1001363.

14. Gurol-Urganci I, de Jongh T, Vodopivec-Jamsek V, Atun R, Car J. Mobile phone messaging reminders for attendance at healthcare appointments. The Cochrane database of systematic reviews. 2013 (12):CD007458. PMID: 24310741. doi: 10.1002/14651858.CD007458.pub3.

15. Guy R, Hocking J, Wand H, Stott S, Ali H, Kaldor J. How effective are short message service reminders at increasing clinic attendance? A meta-analysis and systematic review. Health services research. 2012 Apr;47(2):614-32. PMID: 22091980. doi: 10.1111/j.1475-6773.2011.01342.x.

16. Hall CS, Fottrell E, Wilkinson S, Byass P. Assessing the impact of mHealth interventions in low- and middle-income countries--what has been shown to work? Global health action. 2014;7:25606. PMID: 25361730. doi: 10.3402/gha.v7.25606.

17. Hamine S, Gerth-Guyette E, Faulx D, Green BB, Ginsburg AS. Impact of mHealth chronic disease management on treatment adherence and patient outcomes: a systematic review. Journal of medical Internet research. 2015;17(2):e52. PMID: 25803266. doi: 10.2196/jmir.3951.

18. Krishna S, Boren SA, Balas EA. Healthcare via cell phones: a systematic review. Telemedicine journal and e-health : the official journal of the American Telemedicine Association. 2009 Apr;15(3):231-40. PMID: 19382860. doi: 10.1089/tmj.2008.0099.

19. Liang X, Wang Q, Yang X, Cao J, Chen J, Mo X, et al. Effect of mobile phone intervention for diabetes on glycaemic control: a meta-analysis. Diabetic medicine : a journal of the British Diabetic Association. 2011 Apr;28(4):455-63. PMID: 21392066. doi: 10.1111/j.1464-5491.2010.03180.x.

20. Peiris D, Praveen D, Johnson C, Mogulluru K. Use of mHealth systems and tools for non-communicable diseases in low- and middle-income countries: a systematic review. Journal of cardiovascular translational research. 2014 Nov;7(8):677-91. PMID: 25209729. doi: 10.1007/s12265-014-9581-5.

21. Valles Ortiz PM, Miranda Felix P, García Sosa ES. Mensajes de texto para el control glucémico en adultos con diabetes tipo 2: revisión sistemática. Enfermería Global. 2015;14:435-44.

22. Vodopivec-Jamsek V, de Jongh T, Gurol-Urganci I, Atun R, Car J. Mobile phone messaging for preventive health care. The Cochrane database of systematic reviews. 2012;12:CD007457. PMID: 23235643. doi: 10.1002/14651858.CD007457.pub2.

23. Whittaker R, McRobbie H, Bullen C, Rodgers A, Gu Y. Mobile phone-based interventions for smoking cessation. The Cochrane database of systematic reviews. 2016;4:CD006611. PMID: 27060875. doi: 10.1002/14651858.CD006611.pub4.
